# The Occurrence of Non-Regulated Mycotoxins in Foods: A Systematic Review

**DOI:** 10.3390/toxins15090583

**Published:** 2023-09-20

**Authors:** Octavian Augustin Mihalache, Marthe De Boevre, Luca Dellafiora, Sarah De Saeger, Antonio Moretti, Laetitia Pinson-Gadais, Nadia Ponts, Florence Richard-Forget, Antonia Susca, Chiara Dall’Asta

**Affiliations:** 1 Department of Food and Drug, University of Parma, 43124 Parma, Italy; augustinoctavian.mihalache@unipr.it (O.A.M.); luca.dellafiora@unipr.it (L.D.); 2 Center of Excellence in Mycotoxicology and Public Health, Ghent University, 9000 Ghent, Belgium; marthe.deboevre@ugent.be (M.D.B.); sarah.desaeger@ugent.be (S.D.S.); 3 ISPA-CNR—Institute of Sciences of Food Production, National Research Council, 70126 Bari, Italy; antonio.moretti@ispa.cnr.it (A.M.); antonella.susca@ispa.cnr.it (A.S.); 4 INRAE, UR1264 Mycology and Food Safety (MycSA), F-33882 Villenave d’Ornon, France; laetitia.pinson-gadais@inrae.fr (L.P.-G.); nadia.ponts@inrae.fr (N.P.); florence.forget@inrae.fr (F.R.-F.)

**Keywords:** non-regulated mycotoxins, data quality, occurrence, risk assessment, public health

## Abstract

The aim of this systematic review is to provide an update on the occurrence and co-occurrence of selected non-regulated mycotoxins and provide an overview of current regulations. Fifteen non-regulated mycotoxins were found in 19 food categories worldwide. On top of that, 38 different combinations of non-regulated mycotoxins were found, with mixtures varying from binary combinations up to 12 mycotoxins. Taking into consideration the amount of evidence regarding the prevalence and co-occurrence of non-regulated mycotoxins, future steps should be taken considering continuous monitoring, scientific exchange, and generation of high-quality data. To enhance data quality, guidelines outlining the minimum quality criteria for both occurrence data and metadata are needed. By doing so, we can effectively address concerns related to the toxicity of non-regulated mycotoxins. Furthermore, obtaining more data concerning the co-occurrence of both regulated and non-regulated mycotoxins could aid in supporting multiple chemical risk assessment methodologies. Implementing these steps could bolster food safety measures, promote evidence-based regulations, and ultimately safeguard public health from the potential adverse effects of non-regulated mycotoxins.

## 1. Introduction

Mycotoxins are toxic secondary metabolites produced by fungi that are capable of contaminating a wide range of food commodities, including grains, nuts, fruits, and vegetables [1]. Food contamination with mycotoxins can occur through crops infected with fungi producing toxins in the field or during various stages of the food chain, such as storage, transportation, and food processing [2,3,4]. Mycotoxins are present throughout the cereal food chain and can cause various acute and/or chronic health problems such as immunosuppression, carcinogenesis, endocrine-disrupting activity, gastrointestinal disorders, and kidney damage [5,6,7,8]. Therefore, several countries have implemented regulations to limit mycotoxin exposure through food and minimize their levels as much as possible. In Europe, the EC (European Commission) has established different maximum limits for mycotoxin exposure in adults and children through Commission Regulation (EC) No. 915/2023. However, although hundreds of mycotoxins have been described in the literature, only a few of them are regulated based on their widespread occurrence and well-described toxicological effects.

The European Union closely monitors and strictly regulates several mycotoxins, such as aflatoxins (AFB1, B2, G1, G2, and M1), deoxynivalenol (DON), fumonisins (FB1 and FB2), ochratoxin A (OTA), zearalenone (ZEN), and patulin (PAT) [9]. In addition to setting regulatory limits, the EC proposed recommended levels for T-2 and HT-2 toxins (T2; HT-2) in cereals and cereal-based products [10] and for alternariol (AOH), alternariol methyl ether (AME), and tenuazonic acid (TeA) in foods such as cereal-based foods for infants, legumes, and tree nuts [11].

Regulatory limits are set when a risk assessment process indicates a health concern, i.e., when exposure for a certain population is close to the health-based guidance values. For this purpose, a large occurrence dataset and sound toxicological data are needed. Other mycotoxins, such as nivalenol (NIV) and those from the *Alternaria* family, are exempt from regulation or are only partially regulated despite their toxicological relevance. This is mainly due to gaps in occurrence data, although recent reports indicate an increasing prevalence due to climate change. Other mycotoxins, such as enniatins (ENNs) and beauvericin (BEA), are acknowledged to widely occur in crops, but their toxicological relevance still needs to be consistently ascertained.

Non-regulated mycotoxins, whose prevalence in food and feed commodities is increasing due to environmental factors, i.e., climate change, are often referred to as emerging mycotoxins [12]. These mycotoxins have been found in a variety of foods, including cereals, nuts, spices, and processed foods. A typical example of a non-regulated mycotoxin that requires monitoring is sterigmatocystin (STC), which serves as a precursor of aflatoxin biosynthesis and is structurally related to aflatoxin. Although STC is characterized by a low level of acute toxicity, the main concern results from its carcinogenic nature, which is roughly one-tenth that of aflatoxin B1 [13,14].

In recent years, advances in analytical methodologies, mainly due to high-resolution mass spectrometry (HRMS) instrumentation becoming more affordable for control laboratories, have allowed the screening of a larger spectrum of compounds in a larger set of commodities beyond regulated ones. This has ultimately led to an increasing interest in the occurrence of non-regulated mycotoxins in food and feed. Unfortunately, a lack of analytical standards and reference materials still represents a significant hurdle in the collection of high-quality occurrence data.

In the context of mycotoxin contamination, changes in climate, such as drought and increased temperature levels, lead to new challenges [15,16]. In response to global warming, some fungal species might shift their geographical distribution, leading to a significant change in the pattern of mycotoxin occurring in crops [16]. Therefore, the increasing occurrence of mycotoxins due to climate change might lead, in some cases, to a reconsideration of maximum limits (MLs). In this direction, monitoring the occurrence of non-regulated mycotoxins with the highest dietary exposure is essential to assess if and which mycotoxin poses a human risk.

Therefore, the objectives of this systematic review are:

- To provide an update on occurrence data for non-regulated mycotoxins in foods with the highest dietary exposure (according to data retrieved from the European Food Safety Authority);
- To provide an overview of the current regulations and highlight any gaps in data related to these issues;
- Highlight the need for high-quality occurrence and metadata in risk assessments.

## 2. Results and Discussions

### 2.1. Systematic Review Process

**Figure 1:**
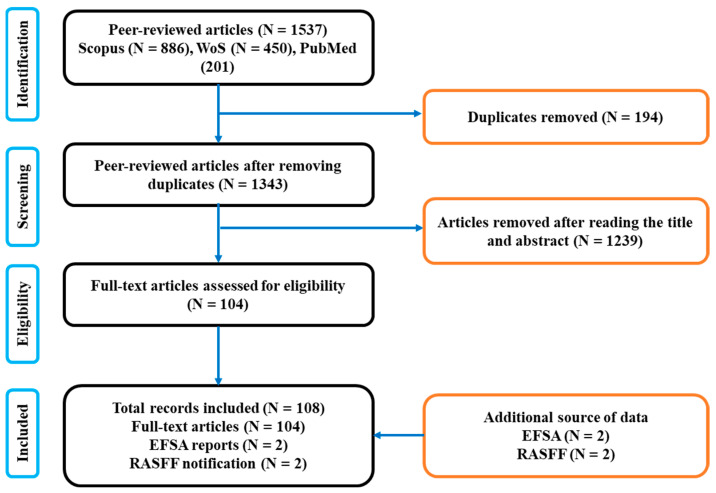
Flowchart outlining the studies that were included and excluded based on the PRISMA guideline; EFSA = European Food Safety Authority; RASFF = Rapid Alert System for Food and Feed; WoS = Web of Science.

Among the 2624 articles published between 2018 and 2023, 1537 were retrieved from Scopus, 886 from Web of Science (WoS), and 201 from PubMed. Following an initial assessment, a total of 194 duplicate records were identified and removed. Additionally, during the screening of titles and abstracts, another 1237 records were excluded from the review due to various reasons, such as focusing on feed rather than food or lacking sufficient detail regarding data. Upon assessing the eligibility of the remaining records, a total of 108 were deemed suitable for inclusion in the present systematic review (Figure 1).

To compensate for the scarcity of data related to non-regulated mycotoxins, we included in our study relevant reports and notifications from the European Food Safety Authority (EFSA) and the Rapid Alert System for Food and Feed (RASFF).

### 2.2. Characteristics of the Articles Included in the Review

Our review has three sources of data, including 104 scientific articles with 203 studies from 35 countries, two reports from EFSA, and two notifications from RASFF reported by Switzerland and Germany. Table S1 provides all the data regarding the region, food, the analytical method used, and the mean/range of the contamination level.

**Figure 2:**
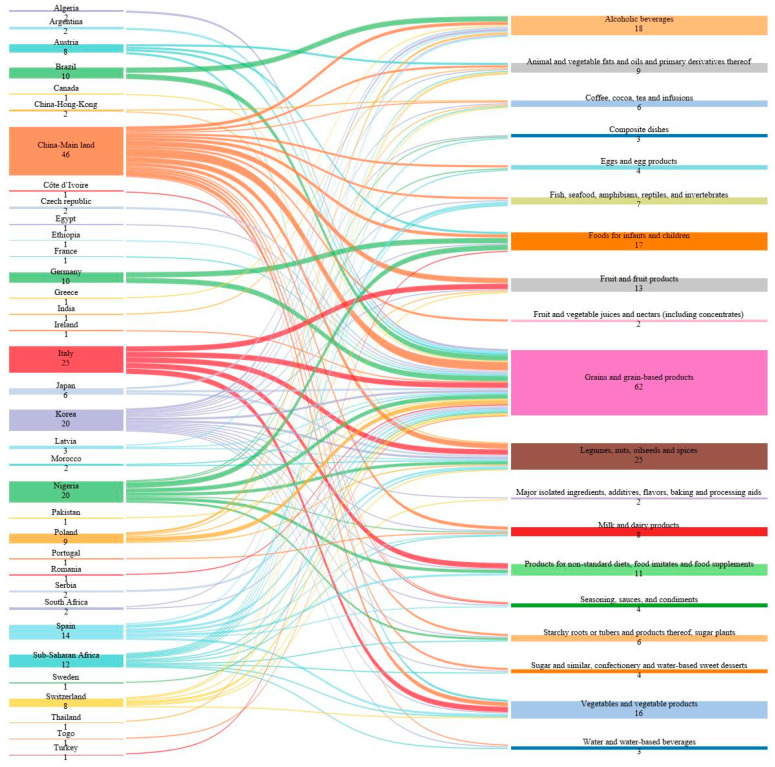
Number of studies from each country/region per type of food; wider nodes represent a higher number of studies for each type of food that was analyzed.

As shown in Figure 2, most of the studies were from China (46/203; 22.7%), Italy (25/203; 12.3%), Nigeria (20/203; 9.9%), and South Korea (20/203; 9.9%), and Spain (14/203; 6.9%), while the lowest number were retrieved from Portugal (1/203; 0.5%), Romania (1/203; 0.5%), and Sweden (1/203; 0.5%). These data show the geographical distribution of where there was a higher interest in the occurrence of non-emerging mycotoxins and also where more research is needed.

**Figure 3:**
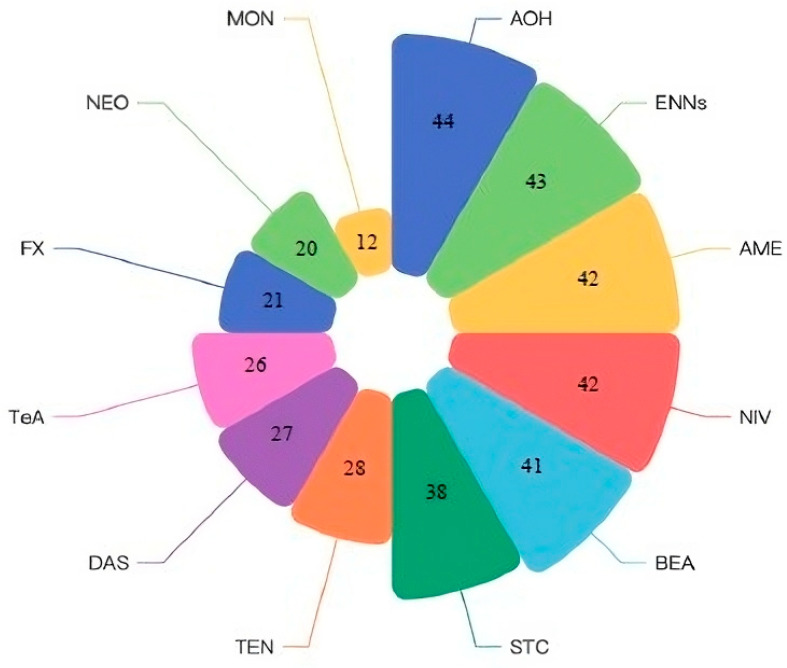
Records included for each non-regulated mycotoxin; AOH = alternariol; AME = alternariol methyl ether: TeA = tenuaszonic acid; TEN = tentoxin; BEA = besauvericin; ENNs = enniatins; DAS = diacetoxyscirpenol; NIV = nivalenol; FX = fusarenon X; NEO = neosolaniol; STC = sterigmatocystin; MON = moniliformin.

The largest proportion of studies were about mycotoxins produced by *Alternaria* spp. such AOH (N = 44/203; 21.7%) and AME (N = 42/203; 20.7%) followed by mycotoxins from the *Fusarium* genera like ENNs (N = 43/203; 21.2%), NIV (42/203; 20.7%) and beauvericin (BEA) (N = 41/203; 20.2%) (Figure 3). A lower number of studies focused on neosolaniol (NEO) (N = 20/203; 9.9%) and moniliformin (MON) (N = 12/203; 5.9%), both produced by species of the *Fusarium* genera. The reports from EFSA provided data regarding diacetoxyscirpenol (DAS) and MON, while notifications from RASFF were related to AOH and TeA.

**Figure 4:**
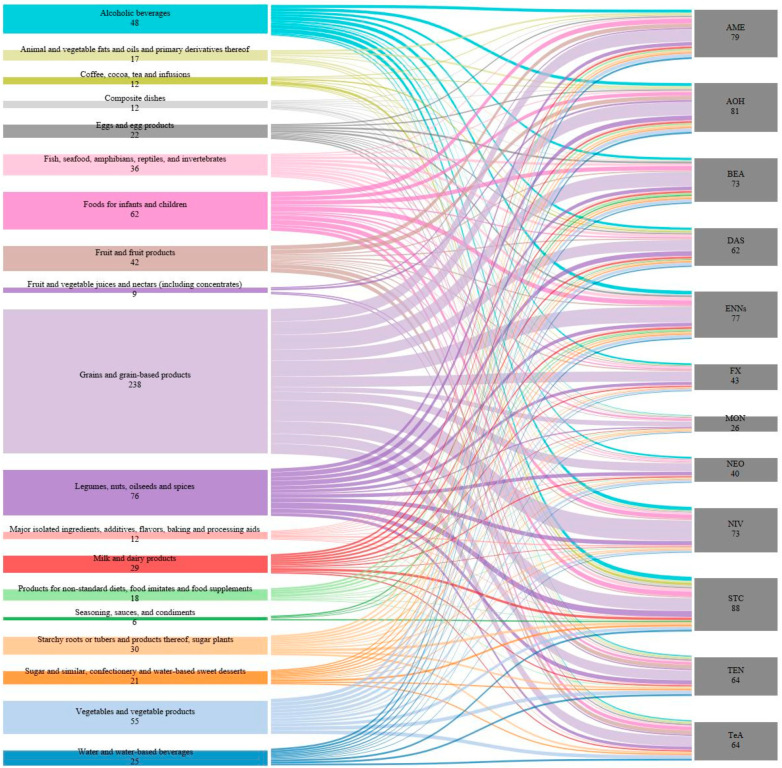
Total number of times each food was analyzed and the target mycotoxins that were found in each food; wider nodes represent a higher number of studies.

Figure 4 displays the number of studies with analyzed food and the target mycotoxins for each food. Grains and grain-based products (i.e., wheat, rice, maize, and flours) were the most extensively studied food with 15 non-regulated mycotoxins analyzed including AOH, AME, TEN, TeA, BEA, ENNs (ENNA, ENNA1, ENNB, ENNB1), MON, NEO, NIV, STC, fusarenon X (F-X), and DAS. Understanding the presence and levels of these mycotoxins is crucial for maintaining food quality and safety, especially considering their potential health implications if their exposure is high. Legumes, nuts, oilseeds, spices (i.e., pulses), and food for infants and children (cereal based-foods, infant formulae, etc.) were also studied extensively. On the other hand, coffee, cocoa, tea, infusions, composite dishes (i.e., granola, popcorn, instant food, etc.), fruit and vegetable juices, and nectars (orange juice, apple juice, etc.) were among the least studied foods for the occurrence of non-regulated mycotoxins.

Grains and products thereof are among the most studied commodities/foods for the occurrence of both regulated and non-regulated mycotoxins worldwide.

### 2.3. Worldwide Occurrence of Non-Regulated Mycotoxins in 2018–2022

Table 1 reports the mean concentration/range of values for non-regulated mycotoxins pooled from their worldwide occurrence in 2018–2022 for 19 food categories.

In grains and grain-based products, all 15 non-regulated mycotoxins were reported. The highest concentration values were for NIV (4175 μg/kg) and ENNs, specifically ENB (2667 μg/kg) and ENB1 (1449 μg/kg). Although at very low concentrations, a mean of 2.07 μg/kg and 0.93 μg/kg was retrieved for STC and NEO, respectively. Grains also had the highest contamination values out of all the food groups for AOH and AME, reaching 1689 μg/kg and 6812 μg/kg in barley from Argentina [17]; TEA had a value as high as 92,002 μg/kg in wheat, which was also from Argentina [18]. Additionally, the highest concentrations of ENA1, ENB, ENB1, BEA, MON, DAS, NIV, STC, and FX levels were reported in grains and grain-based products, ranging from 70 μg/kg for DAS in grains from Japan [19] to 13,335 μg/kg for ENNB in triticale cultivated in France [20].

Vegetables and vegetable products were positive for 10 mycotoxins, among which TeA had the highest mean contamination value (548 μg/kg), while the lowest value was associated with NEO (0.52 μg/kg). Meanwhile, starchy roots or tubers and products thereof had very low contamination values varying from 0.16 μg/kg ENA1 to 2.58 μg/kg BEA.

Legumes, nuts, oilseeds, and spices recorded the highest pooled mean value of TeA (4578 μg/kg), while the lowest value was for DAS (0.22 μg/kg). Legumes, specifically edible sunflower seeds, also had the highest TEN contamination value at 570 μg/kg in Italy [21]. Fruit and fruit products were mainly contaminated with mycotoxins from the *Alternaria* genera, such as AOH, AME, TeA, and TEN, with values between 0.98 and 366 μg/kg. Also, fruit, vegetable juices, and nectars (including concentrates) were contaminated with AOH and TeA but at much lower levels (1.71 μg/kg and 4.26 μg/kg, respectively).

The contamination of milk and dairy products with non-regulated mycotoxins such as ENNs, BEA, AME, and TeA was reported. The contamination values were low, ranging from 0.16 μg/kg for BEA to 2.53 μg/kg for TeA [22,23].

Sugar and similar confectionery and water-based sweet desserts were the least contaminated food group, with only AME, TEN, and FX being detected. In this category, we included sugar and sugar products, aquatic foods, and aquatic food products from total diet studies [22,23] with pooled mean values of 0.12 μg/kg AME, 0.12 μg/kg TEN, and 6.92 μg/kg FX. However, used items/ingredients in these food categories are not mentioned, making it difficult to trace their origin of contamination with AME, TEN, and FX.

Coffee, cocoa, tea, and infusions were contaminated with mycotoxins from the *Alternaria* genera, although at a very low level with STC (0.08 μg/kg). Meanwhile, alcoholic beverages had mean pooled contamination values ranging from 0.16 μg/kg for ENA to 23.3 μg/kg for AOH (apple cider, wine, and beer) [23,24]. This group also recorded the highest NEO contamination level, which reached 21 μg/kg in an alcoholic beverage from South Africa (beer made from maize) [25].

Non-regulated mycotoxins could occur in all considered categories of infant food (i.e., infant formulae, cereal-based foods, crackers, and noodles for children).

Only DAS and FX were not detected in these products. The pooled contamination levels ranged from 0.4 μg/kg AOH (cereal-based cereals for infants and children from Germany) [26] to 64.2 μg/kg MON [27,28,29,30] (infant food from Nigeria).

It is important to highlight that AOH, AME, TeA, ENNs, BEA, and MON may also occur in products pertaining to the group for non-standard diets, food imitates, and food supplements. The mean range of values varied from 0.21 μg/kg AOH to 323 μg/kg ENNA. Also, the highest ENNA concentration was recorded in soy-based burgers from Italy (632 μg/kg) [31]. A recent study also reported emerging mycotoxins such as AOH, AME, and TEN in plant-based meat alternatives ranging from 0.3 μg/kg AME to 12.1 μg/kg AOH [32].

Recently, the European Commission set up indicative levels for AOH, AME, and TeA in eight food products, including processed tomato products, paprika powder, sesame and sunflower seeds, sunflower oil, tree nuts, dried figs, and cereal-based foods for infants and young children [11].

Data from the literature clearly shed light on the distribution of non-regulated mycotoxins worldwide, indicating that such compounds might occur in many food products, including grains, vegetables, starchy roots, legumes, nuts, fruits, milk, and dairy products, sugar and similar confectionery, coffee, cocoa, tea, and alcoholic beverages. However, to support risk assessments and allow the regulator to correctly manage the risk posed by not-yet-regulated compounds, the abovementioned mycotoxins should be included in routinary monitoring plans for grains, vegetables, and legumes to collect occurrence data that are well distributed over time and geography.

### 2.4. Worldwide Co-Occurrence of Non-Regulated Mycotoxins and Potential Health Effects

Data related to the worldwide co-occurrence of non-regulated mycotoxins are gathered in Supplementary Figures S1 and S2. Mycotoxin co-occurrence in food can lead to either additive, synergistic, or antagonistic toxic effects, the former amplifying the potential adverse impact on consumers’ health [33]. Although the overall effect of a mycotoxin mixture in vivo remains largely unknown, the recent literature has discussed their potentially combined action in vitro, with most cases reporting additive or synergistic effects [34,35,36].

We have found 38 different combinations of two mycotoxins, 20 combinations of three mycotoxins, 13 combinations of four mycotoxins, 12 combinations of five and six mycotoxins, 8 combinations of seven mycotoxins, and 9 mixtures of >seven mycotoxins (Figures S1 and S2). The highest number of two-mycotoxin combinations was for ENNs and *Alternaria* toxins such as ENNA + ENNB1 (60 times), BEA + ENNB (56 times), AME + TeA (53 times), AME + AOH (53 times). The major contributors were food products for infants and children (infant noodles, infant crackers, infant puree food, etc.) and grains and grain-based products (wheat, barley, wheat flour, etc.). The least co-occurring two-mycotoxin co-combination was that of DAS + NEO (2 times), BEA + FX (2 times), and BEA + DAS (2 times), mostly in grains and grain-based products.

For three-mycotoxins combinations, the most co-occurring ones were ENB + ENB1 + BEA (48 times), ENA1 + ENB + ENB1 (46 times), AME + TeA + TEN (41 times), AOH + AME + TEA (39 times). These mixtures were mostly found in grains and grain-based products and food imitates (i.e., oat beverages, rice beverages, soy beverages, etc.). The least co-occurring mixtures were those of DAS + NIV + STC (2 times) and ENA + DAS + NEO (1 time), mostly in grains and grain-based products.

The mixtures of four mycotoxins consisted mainly of ENA1 + ENB + ENB1 + BEA and ENA + ENA1 + ENB + ENB1 (37 times) and ENA + ENB + ENB1 + BEA (32 times) in grains and grain-based products, food imitates, and foods for infants and children. The lowest frequency was for ENA1 + ENB1 + BEA + FX and AOH + AME + NIV + NEO (two times), which were found in grains and grain-based products.

ENNs and BEA are often found together, as indicated by the prevalence of the mixtures of ENA + ENA1 + ENB + ENB1 + BEA, which were found 30 times mostly in grains and grain-based products, food imitates, and foods for infants and children. Another combination of six mycotoxins was reported, ENA1 + ENB + BEA + NIV + NEO. This mixture was, however, only observed once in wheat.

ENNs and BEA often occur in combination with *Alternaria* toxins, as indicated by the mix AME + TeA + TEN + ENB + ENB1 + BEA and AME + ENA + ENA1 + ENB + ENB1 + BEA, which was found 14 times in grains and grain-based products, in foods for infants and children, vegetables and vegetable products, and legumes, nuts, oilseeds, and spices. Surprisingly, these mycotoxins were found once in the same mixture with STC, NEO, and FX (AOH + AME + ENB + STC + NEO + FX); this was the same for wheat flour.

Combinations of all ENNs with BEA and *Alternaria* mycotoxins were also reported (AME + TEN + ENA + ENA1 + ENB + ENB1 + BEA) 11 times in foods such as legumes, nuts, oilseeds, spices and grains, and grain-based products. Meanwhile, AOH + AME + TEN + ENA + ENA1 + ENB + ENB1 was reported only once in oat flakes.

The highest number of co-occurring mycotoxins was 12 mycotoxins in the form of the mixture: AOH + AME + TeA + TEN + ENA1 + ENB + ENB1 + BEA + MON + DAS + NIV + STC found in sorghum from Ethiopia [37].

The different mixtures described in the studies encompassed by this review highlight the need to further assess combined toxic effects as there could be an amplification of effects; therefore, group-based risk assessments and MLs should be considered.

**Figure 5:**
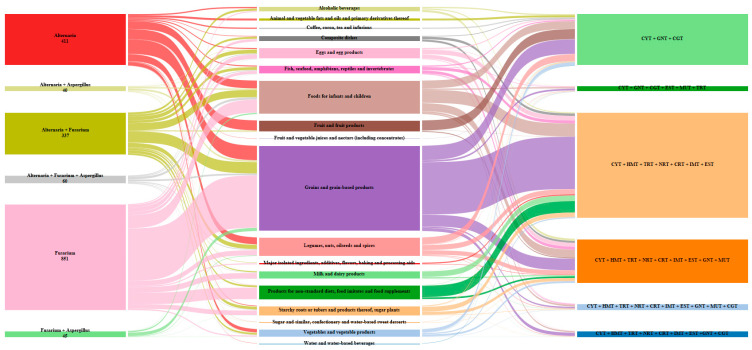
Fungi genera and/or mixtures of fungi genera: *Alternaria*, *Aspergillus*, and *Fusarium* correlated with the food contributors and the endpoints related to the type of fungi; cytotoxicity (CYT), haematotoxicity (HMT), teratogenicity (TRT), neurotoxicity (NRT), cardiotoxicity (CRT), immunotoxicity (IMT), estrogenicity (EST), genotoxicity (GNT), mutagenicity (MUT), and carcinogenicity (CGT).

Based on data from the literature and food safety authorities such as EFSA, we identified the following endpoints for *Alternaria, Aspergillus,* and *Fusarium* fungi: cytotoxicity (CYT), haematotoxicity (HMT), teratogenicity (TRT), neurotoxicity (NRT), cardiotoxicity (CRT), immunotoxicity (IMT), estrogenicity (EST), genotoxicity (GNT), mutagenicity (MUT), and carcinogenicity (CGT) [5,6,14,38,39,40,41]. The number of mycotoxin mixtures according to the fungi genera, the main contributors, and endpoints are shown in Figure 5. The following mixtures were found: mixtures produced by *Alternaria* fungi occurred 411 times, *Alternaria + Aspergillus* fungi 40 times, *Alternaria + Fusarium* 337 times, *Alternaria + Fusarium + Aspergillus* 60 times, mixtures of *Fusarium* 851 times, and *Fusarium + Aspergillus* 45 times. This reveals the co-occurrence of non-emerging mycotoxins from different fungi with different toxic effects, most of which have additive and/or synergistic interactions.

According to Ficheux et al. [42], exposing hematopoietic progenitors, which are cells found in both the blood and bone marrow, to BEA + ENB results in additive myelotoxic effects. Trichothecenes’ main targets are hematopoietic progenitors, specifically the Colony-Forming Unit-Granulocyte and Macrophage (CFU-GM), Colony-Forming Unit-Megakaryocyte (CFU-MK), and Burst-Forming Unit-Erythroid (BFU-E), which are responsible for producing human white blood cells, platelets, and red blood cells, respectively [43].

Based on their myelotoxicity in vitro and regarding the lowest dose that does not affect hematopoietic progenitor proliferation, regardless of their lineage, the following mycotoxins were classified as follows: HT-2 > T-2 > DAS > DON, BEA > MON > ENB > OTA. Even though BEA myelotoxicity is comparable to some trichothecenes [42], it is still not regulated.

In vitro results demonstrate that AOH and AME can trigger apoptosis via a PTP-dependent mechanism when tested individually, and their combination has an additive effect, leading to a considerable increase in cell mortality when tested on HCT116 cells (human intestinal cell line) [44]. AOH and AME trigger the permeabilization of mitochondrial membranes, which is regarded as the stage where apoptosis becomes irreversible [45,46].

Similar results were reported by den Hollander et al. [47], who showed that the combination of AOH + AME and AME + TeA had an increased cytotoxic effect on liver cancer cells (HepG2) and human colon carcinoma cells (Caco-2 cells) when compared with the single exposition of these mycotoxins. Except for high concentrations, the mixture of AOH + AME + TeA does not exert higher cytotoxic effects on Caco-2 cells and HepG2 cells when compared with their binary combinations [47].

These two-mycotoxin combinations of NIV + FX are also concerning due to the fact that no matter the toxicity level, this combination has an additive interaction, increasing the toxic effect on Caco-2 cells [48].

BEA often co-occurs in food products with OTA, especially in cereals. A recent study investigated the cytotoxicity of OTA and BEA in HepG2 cells, both individually and in combination, and the results showed that synergism and additive effects were present in the binary mixtures [35].

ENNs are known to reduce cell viability in human colon cell lines (HT-29 and Caco-2 cells) and in human liver cells (Hep-G2 cells) with ENA1 being the most toxic [49]. The combined impact of ENNs results in a substantial decrease in the integrity of the intestinal barrier in IPEC-J2 cells [36] and, at high concentrations, ENNs exhibit synergistic and cytotoxic effects on CHO-K1 cells, which are derived from the Chinese hamster ovary cell line [50]. Taking into account how often ENNs co-occur and that the interactions between ENNs are additive or synergistic at higher concentrations [33], this is an issue that requires more investigation and needs to be addressed at a legislative level. Placing maximum limits on ENNs could reduce dietary exposure and, consequently, potential human health risks.

Depending on the mycotoxins that co-occur and the studied cell line, interactions can also be antagonistic. For example, the binary combinations of ENNB, DON, NIV, and ZEN result in antagonistic effects in the Caco-2 colorectal carcinoma cell line [51]. The binary combination of each of these mycotoxins, with TeA results in a further decrease in the toxicity level [51]. At the same time, a synergistic effect was observed for ENNB + AOH, leading to a decreased cell viability for Caco-2 cells [34].

While more in vitro studies are needed to elucidate the combined toxic effects of non-regulated mycotoxins, research has hitherto proven that these mycotoxins co-occur frequently in many food products and that most of the time, their interactions are characterized by additive or synergistic effects. Moreover, combined effects of mycotoxins with other food constituents have also been reported, which may significantly modify their nominal potency. In this light, mycotoxins should also be assessed in this sense to better describe their activity in a more realistic context—i.e., within the chemical space they are typically in.

### 2.5. Data Quality in Occurrence Data

One of the primary steps when dealing with non-regulated mycotoxins is to ensure the quality of data. Reliable and accurate data on the occurrence and toxicity of these mycotoxins are crucial for risk assessment and decision-making. This requires standardized sampling and analysis methods, quality control measures, and collaboration between researchers, regulatory agencies, and industry stakeholders. By establishing rigorous data collection and analysis protocols, we can enhance our understanding of non-regulated mycotoxins and their potential risks.

For this purpose, a lack of reference materials is the most urgent hurdle to be overcome to ensure a quick transition from qualitative screening data to accurate determination.

In order to improve data quality, guidelines are needed for the minimum quality criteria for occurrence data and metadata. A lack of standardization, such as inconsistent methodologies and data collection, makes it challenging to compare data and report them to food safety authorities. Occurrence data need to be collected periodically to obtain updated mycotoxin levels and assess if there are any ongoing trends and if regulatory actions are needed, especially considering the climate change scenario.

To effectively manage non-regulated mycotoxins, it is important to establish continuous monitoring programs. These programs could cover various stages of the food supply chain, including cultivation, harvesting, storage, processing, and distribution. This information is required to develop appropriate risk management strategies. For example, the regular testing of crops during cultivation and harvesting can help identify mycotoxin-prone regions or crops, allowing for preventive measures if needed. In storage and processing facilities, continuous monitoring could ensure the early detection of mycotoxin buildup, enabling timely actions such as temperature and humidity control, proper ventilation, and mycotoxin mitigation techniques.

Continuous risk assessments play a vital role in managing non-regulated mycotoxins. To have robust risk assessment methodologies; therefore, more data are needed considering exposure levels, vulnerable populations, and cumulative effects of multiple mycotoxins.

Promoting scientific exchange and collaboration is critical in this framework. By sharing information, findings, and data, researchers, regulatory bodies, and industry stakeholders can enhance knowledge acquisition and risk assessment. International cooperation and data sharing on occurrence and prevalence in different regions is necessary to reach a global perspective view on non-regulated mycotoxins and promote collective efforts in risk mitigation.

Collaboration between countries could enable the pooling of resources and expertise to address mycotoxin-related challenges. Facilitated communication, providing funding, and establishing harmonized guidelines for the management of non-regulated mycotoxins are currently key missing initiatives.

## 3. Study Limitations

Our review is not without limitations. The main limitation of our review is publication bias. Publication bias is the most common issue in systematic reviews and can result in a higher publication frequency of effective studies over ineffective studies [52]. Therefore, the findings presented in this review may overestimate the actual occurrence data of non-regulated mycotoxins in foods. Another limitation is that researchers often report the contamination values for a group of food items without mentioning what items are included in that food category and/or the ingredients. This leads to uncertainties, making it difficult to establish the origin of contamination. The use of different analytical methods with varying limits of detection (LOD) and quantification (LOQ) leads to further limitations, especially for data related to mixtures of mycotoxins. Data harmonization is a key component for the improvement of data quality in dietary exposure and risk assessments [53]. Mycotoxin contamination is dependent on several factors, such as the technological process, storage, and transportation of food. Another limitation is related to the toxic effects of non-regulated mycotoxins. Currently, we do not have enough in vivo toxicity data and, therefore, can only rely on in vitro data.

## 4. Conclusions

This systematic review was a first attempt at highlighting the occurrence/co-occurrence of 15 non-regulated mycotoxins worldwide from 2018 to 2022. Most of the studies focused on the occurrence of non-regulated mycotoxins in grains and grain-based products, legumes, and foods for infants and children. Thirty-eight different mixtures of non-regulated mycotoxins were found, ranging from two mycotoxins up to twelve mycotoxins. The co-occurrence of these mycotoxins and group-based toxicity assessment should be taken into consideration for a proper risk assessment. The current data indicate that most interactions between these mycotoxins lead to additive/synergistic toxic effects, as shown by many in vitro studies. Gathering more data related to the co-occurrence of regulated and non-regulated mycotoxins could facilitate and support multiple chemical risk assessment methodologies [54,55].

Addressing concerns related to non-regulated mycotoxins and their toxicity requires a comprehensive approach. Emphasizing the quality of data, continuous monitoring, scientific exchange, and support for continuous risk assessment is crucial for mitigating potential risks to human health. Implementing these steps could enhance food safety measures, facilitate evidence-based regulations, and ultimately protect public health from the adverse effects of non-regulated mycotoxins.

## 5. Materials and Methods

### 5.1. Search Strategy

To ensure the scientific rigor of this review and minimize potential bias, we adhered to the Preferred Reporting Items for Systematic Reviews and Meta-Analyses (PRISMA) statement protocol for screening titles, abstracts, and full texts [56]. This systematic review has not been registered on PROSPERO as it does not cover human data and, therefore, has no registration number.

A systematic review of the literature was performed in January–February 2023 in three databases (Scopus, WebofScience, and PubMed) within the timeframe of January 2018–December 2022 to provide recent occurrence data regarding the most frequently found non-regulated mycotoxins for which a preliminary assessment from EFSA has already been performed. The focus was on foods with the highest dietary exposure at the European level as reviewed by the Scientific Opinions and Reports from EFSA, such as cereals and cereal-based foods, oilseeds, nuts, fruits, vegetables, wine, sauces, coffees, legumes, beans, and potatoes [5,6,14,38,39]. All the food items were grouped into 19 food groups, as indicated by EFSA. For example, cereals like wheat, oat, etc., and cereal-based foods were grouped into grains and grain-based foods, infant formulas and cereal-based foods for children were grouped into foods for infants and children, and any type of vegetable/vegetable-based product was grouped into vegetables and vegetable products, etc.

The review was conducted using the following search strings: (“Alternaria toxin*” OR “Alternariol*” OR “Alternariol monomethyl ether” OR “Tenuazonic acid” OR “Tentoxin*” OR “Enniatins” OR “Beauvericin” OR “Moniliformin” OR “Diacetoxyscirpenol” OR “Nivalenol” OR “Sterigmatocystin” OR “Neosolaniol” OR “Fusarenon X*) AND (“food*” OR “cereal*” OR “oilseed*” OR “nut*” OR “fruit*” OR “vegetable*” OR “bread*” OR “wine*” OR “sauce*” OR “spicy*” OR “coffee*” OR “legume*” OR “bean*”OR “potato*” OR “strachy*”).

Recently, the European regulation for mycotoxins has been updated for AOH, AME, and TeA in eight food products, such as processed tomato products, paprika powder, sesame and sunflower seeds, sunflower oil, etc. [11]. However, we decided to include them in this review due to the fact that they are known to occur in other foods not included in the current legislation and due to their “emerging” nature.

The search was performed by two reviewers in order to minimize bias. Any discrepancies between the reviewers were noted and resolved through discussion. The reviewers met in person before the screening process to discuss study eligibility by defining inclusion and exclusion criteria.

### 5.2. Inclusion and Exclusion Criteria of Data

The systematic review included publications that satisfied specific criteria, such as having a downloadable full-text paper written in English, being published in a peer-reviewed journal, measuring the prevalence and concentration of mycotoxins, mentioning the analytical methods used, and involving samples of products intended for human consumption. Any study that did not meet these criteria, such as those written in languages other than English, lacking full-text availability, lacking concentration values for contaminants, or reviews without undergoing peer review, were excluded.

Data from scientific reports such as those from the European Food Safety Authority (EFSA), the Food Safety Authority Committee on Toxicity (FSA COT), and notifications from the Rapid Alert System for Food and Feed (RASFF) were also extracted.

### 5.3. Data Extraction

For each publication, we included data such as the first author’s name, study year, the analyzed food, the analytical method used, the total number of samples and incidence of contamination, and the mean or range of contamination (μg/kg). Rayyan (an AI Powered Tool for Systematic Literature Reviews) was used to conduct searches, remove duplicates, and manage title and abstract screening [57].

### 5.4. Statistical Analysis

The statistical analyses were conducted using Microsoft Excel 21 (Microsoft, Redmond, WA, USA) and SPSS Statistics 26 (IBM Software Group, Chicago, IL, USA). The contamination values were presented in the form of their average mean value/average range values pooled from the studies included in this review. By pooling the data from multiple studies and presenting the average mean value and average range values, this review aimed to provide a summarized view of the contamination levels and their variability across the included studies. This can allow readers to gain a better understanding of the overall contamination trends and the range of values observed in the reviewed studies.

## Figures and Tables

**Table 1:** Pooled mean concentration values and mean/range values of non-regulated mycotoxins based on worldwide occurrence in 2018–2022.

Mean Contamination ^1^ and (or) Mean Range ^2^ μg/kg(L)	*Alternaria*				ENNs				BEA	MON	DAS	**NIV**	**STC**	**NEO**	**FX**
	AOH	AME	TeA	TEN	ENNA	ENNA1	ENNB	ENNB1							
Grains and grain-based products	118.49	101.77	1178.59	2.5	6.78	12.32	118.41	37.78	47.53	231.58	7.49	70.7	2.07	0.93	80.68
	1.22–36.08	0.73–38.79	2.62–306.06	0.54–10.72	0.81–176.92	0.51–574.68	0.31–2667.24	0.38–1449	0.39–1.86			53.35–4175.7	0.02–0.04	0.26–50	2.8–50
Vegetables and vegetable products	4.17	2.7	73.83	3.13	4.1		1.06	0.73	7.74	ND	ND	ND	0.09	0.52	ND
	2–65.3		5.10–548.36	1.09–1.97			0.16–0.38		15–57				0.09–0.17		
Starchy roots or tubers and products thereof	ND	0.37		0.49		0.16				ND	ND	ND	0.23	ND	ND
		0.11–2.41	0.2–0.41		0.15–1.07		0.21–1.63	0.12–0.44	1.21–2.58				0.10–0.13		
Legumes, nuts, oilseeds and spices	121.47	13.98	1399.21	64.47	1.15	0.25	0.28	0.32	0.78	5.61	0.22	25.74	3.61	1.43	ND
	4.70–93.27	5.50–29.67	27.79–4678.62	4.17–42.6	0.26–1.26	0.22–0.29	1.54–6.49	0.14–0.73	0.32–5.46				0.07–1.37		
Fruit and fruit products	2.95	1.32	44.3	0.98			044		0.71	ND	ND	ND	ND	ND	ND
	1.05–43.68	2–47.8	5.13–365.99	1.05–7.52											
Fish, seafood, amphibians, reptiles and invertebrates	ND	0.38	0.98		3.17	0.20				ND	3.71	ND	0.04	0.21	ND
		0.17–0.38		0.38–0.68			0.18–2.77	0.22–0.89	0.32–1.02						
Milk and dairy products	ND	2.38		ND	1.16; liquid: 1.13	1.61; liquid: 0.77	1.73; liquid: 0.63	1.73; liquid: 0.76	3.84; liquid: 0.52	ND	ND	ND	1.9	ND	ND
			1.75–2.53						0.16–6.31						
Eggs and egg products	ND	0.72		7.01					1.8	ND	ND	ND		ND	ND
		0.72–1.31	0.26–27.73		0.29–1.55	0.27–0.51	0.11–2.18	0.14–0.58	0.78–6.7						
Sugar and similar confectionery and water-based sweet desserts	ND	0.12	ND	0.12	ND	ND	ND	ND	ND	ND	ND	ND	ND	ND	6.92
		
Animal and vegetable fats and oils and primary derivatives thereof	231.83	78.03	19.75	0.78	-	-	-	-	0.3	ND	ND	-	2.92	-	-
		2–2.4		2–3.9											
Fruit and vegetable juices and nectars (including concentrates)	1.71	ND	4.26	ND	-	-	-	-	-	-	-	-	-	-	-
	
Water and water-based beverages	ND	0.16	0.58 ng/L	0.51 ng/L						ND	ND	ND	ND	ND	ND
		
Alcoholic beverages	23.29	21.03			0.16		13.8	0.62	2.24	ND	ND	4.62	18	14.82	167
			2.26–17.62	0.15–0.67											
Coffee, cocoa, tea and infusions			11.5		-	-	-	-	-	-	ND	ND	0.08	-	ND
	<3	<1.3		<1.3									0.13–4.48		
Foods for infants and children	1.26	0.58; breast milk: 3.98 ng/L	22.7	0.96	2.13; breast milk: 0.5 ng/L	0.53; breast milk: 0.9 ng/L	1.83; breast milk: 4.38 ng/L	0.93; breast milk: 0.56 ng/L	1.85; breast milk: 3.88 ng/L	64.24	ND	17.65	0.16; breast milk: 1.2 ng/L	-	ND
	0.4–2						breast milk: 4–9 ng/L		breast milk: 11–19 ng/L			16–19.5	0.1–0.2		
Products for non-standard diets, food imitates and food supplements	92.6; liquid: 0.21	207.5	ND	liquid: 21.3	323.8; liquid: 1.64	67.3; liquid: 1.53	138.4; liquid: 13.40	121.2; liquid: 8.22	liquid: 1.4	11.2	ND	ND		ND	-
								
Composite dishes	2.1	ND	114	ND	ND	ND	ND	ND	4.4	165	ND	16.4	0.43	-	-
					
Seasoning, sauces and condiments	-	-	-	-	ND	ND	ND	12.0	37.9	-	ND	-	0.08	-	-
		
Major isolated ingredients, additives, flavors, baking and processing aids	ND	ND	ND	ND	ND	ND	ND	ND	ND	-	ND	ND	ND	ND	ND

^1^: mean contamination value; ^2^: mean contamination range for the studies which do not provide the mean value and the original data; ND: not detected.; -: not analyzed; blank spaces—if a mean value was provided then the cells for the contamination range values were left in blank and vice versa; AOH = alternariol; AME = alternariol methyl ether: TeA = tenuaszonic acid; TEN = tentoxin; BEA = besauvericin; ENNs = enniatins; DAS = diacetoxyscirpenol; NIV = nivalenol; FX = fusarenon X; NEO = neosolaniol; STC = sterigmatocystin; MON = moniliformin.

## Data Availability

All of the data are available in the Supplementary Materials.

## Supplementary Materials

The following supporting information can be downloaded at: https://www.mdpi.com/article/10.3390/toxins15090583/s1, Figure S1. Worldwide co-occurrence of mixtures of 2–4 non-regulated mycotoxins; wider nodes represent a higher incidence, Figure S2. Worldwide co-occurrence of mixtures of 5–12 non-regulated mycotoxins; wider nodes represent a higher incidence, Table S1. Studies included in the systematic review with foods, region, analytical method, mycotoxins, samples, incidence, contamination values and reference.
