# Supplementary material for: The Occurrence of Non-Regulated Mycotoxins in Foods: A Systematic Review
Source: Toxins (Basel). 2023 Sep 20;15(9):583. doi: 10.3390/toxins15090583 (PMC10534703; doi:10.3390/toxins15090583)
Supplement: Supplementary file 1 [file toxins-15-00583-s001.zip › toxins-2600812supp-figs.pdf]

Supplementary Materials: Occurrence of non-regulated mycotoxins in foods: A systematic review

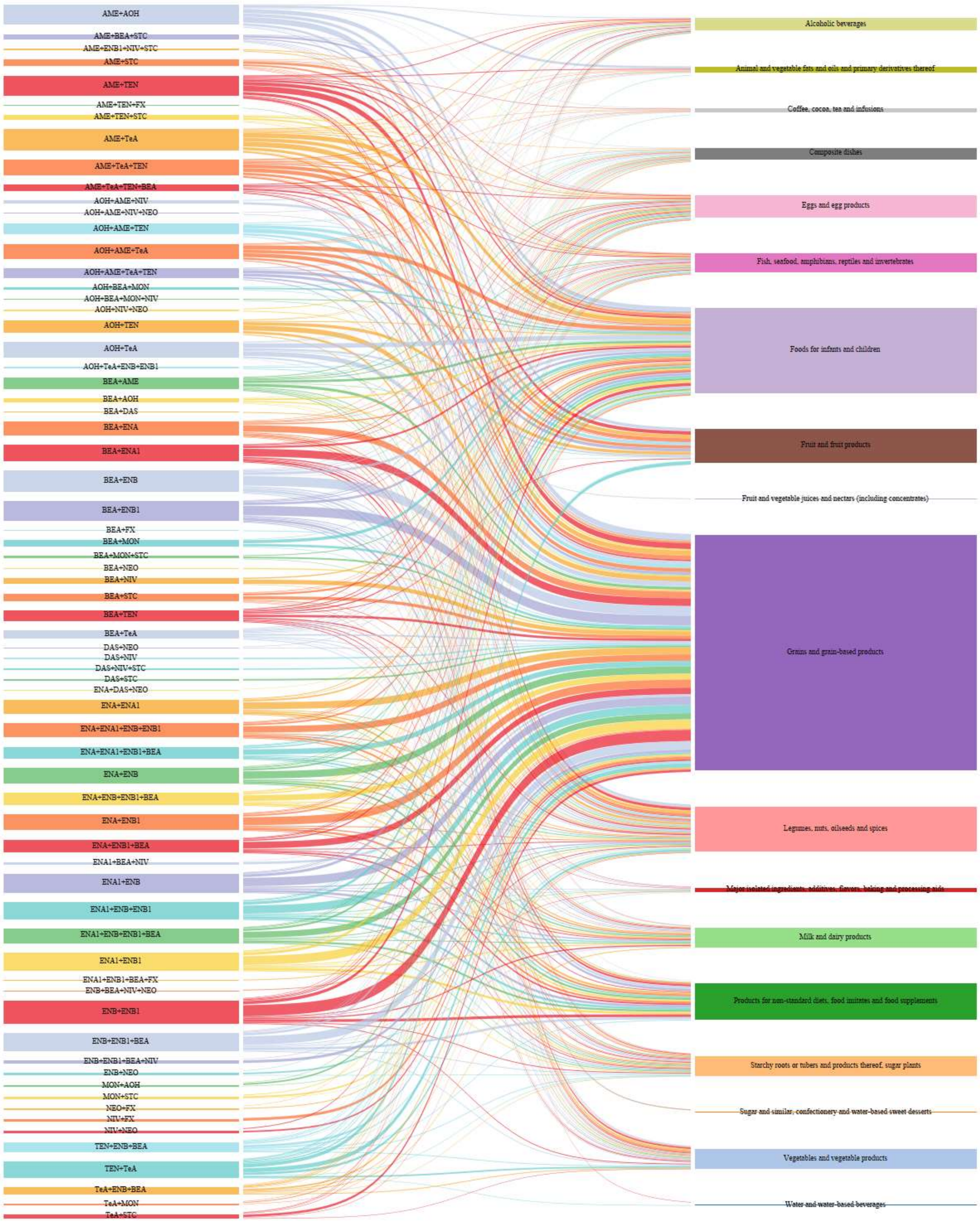

Figure S1. Worldwide co-occurrence of mixtures of 2 – 4 non-regulated mycotoxins; wider nodes represent a higher incidence.

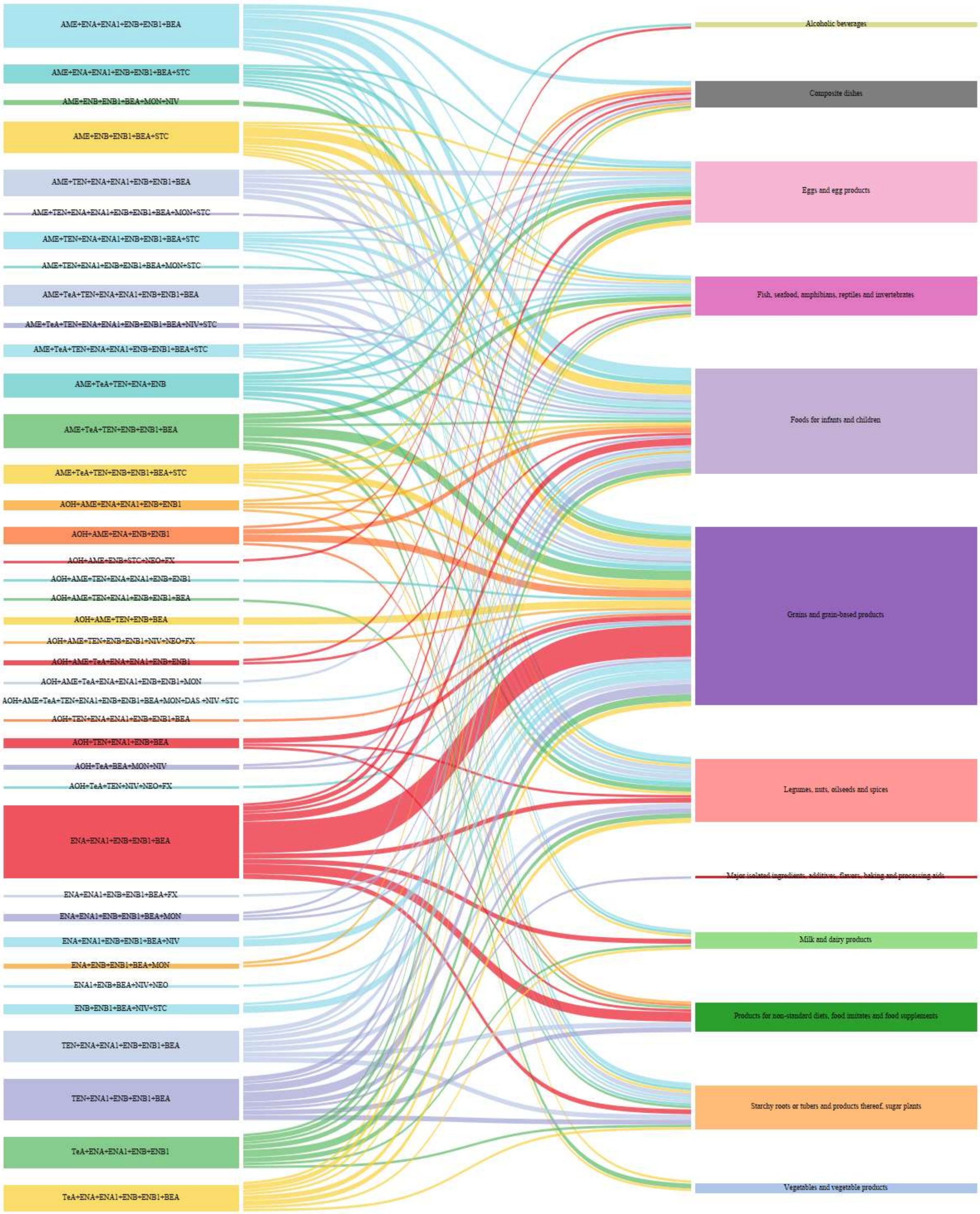

Figure S2. Worldwide co-occurrence of mixtures of 5 – 12 non-regulated mycotoxins; wider nodes represent a higher incidence.
