# Supplementary material for: The Occurrence of Non-Regulated Mycotoxins in Foods: A Systematic Review
Source: Toxins (Basel). 2023 Sep 20;15(9):583. doi: 10.3390/toxins15090583 (PMC10534703; doi:10.3390/toxins15090583)
Supplement: Supplementary file 1 [file toxins-15-00583-s001.zip › toxins-2600812supp-table.pdf]

Supplementary Materials: Occurrence of non-regulated mycotoxins in foods: A systematic review

Table S1. Studies included in the systematic review with foods, region, analytical method, mycotoxins, samples, incidence, contamination values and reference.

Food item	Region/Country	Analytical method	Mycotoxin	No. of samples	Incidence (%)	Mean/Range (Mg/Kg)	Reference
**Kunu (Cereals/Nuts-Based Beverage),**	Nigeria	LC-MS/MS	AME AOH	3	AOH:0	AOH: ND; AME:<2	[1]
			BEA			BEA:<2	
			MON		0	ND	
**Corn By-Products Produced By Wet-Milling**	Korea	LC-MS/MS	NIV	CORN GLUTEN (N = 6)		<LOQ[A]	[2]
				STARCH CORN FROM US,N=6)		4.0 ± 9.9	
				STARCH (CORN FROM EU, N = 2)		ND	
				CORN GERM (N = 6)		<LOQ	
				CORN BRAN (N = 6)		3.8 ± 9.3	
				CORN US (N = 6)		<LOQ	
				CORN EU (N = 2)		ND	
**Winter Wheat**	Poland	LC-UV	NIV	92	70	35.0	[3]
**Grain Barley**	Argentina	HPLC-UV/VIS	NIV	119	29	2.36	[4]
**Latvian Beers**	Latvia	HPLC-HRMS	BEA	25	0	ND	[5]
			ENN		20 (ENNB, ENNB1)	0.05-1.56 MG/L	
			STC		0	ND	
**Wheat Flour**	Japan	LC-MS/MS	NEO DAS	101	0	ND[A]	[6]
**Job's Tears Products**				46	DAS: 63; NEO:13	DAS: 13 NEO: 0.2	

**Rye Flour**				21	0	ND	
**Corn Flour**				27	DAS: 15	DAS: 0.07	
**Azuki Bean**				33	DAS: 9	DAS: 0.02	
**Roasted Coffee Beans**	Hong Kong	UPLC-MS/MS	STC	12	8.3	0.079	[7]
**Black Pepper**				6	50	0.086-0.87	
**Egg**	China	UPLC-MS / MS	ENN	114	0	ND	[8]
			BEA		19.3	1.80	
**Arecae Semen And Its Processed Products**	China	UPLC-MS/MS	DAS	75	0	ND	[9]
			STC		6.7	<LOQ-2.17	
			NEO		0	ND	
**Grape Wine**	Greece	Q-TOF MS-MS	AME AOH	75	0	ND	[10]
**Maize**	Serbia	HPLC–DAD	MON	10	100	1649.3	[11]
						1691.6	
**Grape**	China	UHPLC-MS/MS	AME AOH TEA TEN	56	AME:3.6	0.11-0.15	[12]
					AOH:26.8	0.09–7.15	
					TEA: 28.6	0.25–46.97	
					TEN: 37.5	0.10–1.64	
**Barley And Malting Barley**	Germany	LC–MS/MS	AME	50	(BARLEY) 18 (MALT) 26		[13]
			AOH		26 46		
			TEA		84 96		
			TEN		58 58		
**Beer**	Spain	GC-MS/MS LC-MS/MS	DAS FX ENN BEA STC NEO NIV AOH AME	40	AOH: 90; NEO: 20; NIV: 30	AOH: 19.39MG/L; NEO: 14.67; NIV: 10.01	[14]
**Wine**				40	AOH: 52; AME: 50; NEO: 22; NIV: 12	AOH: 5.23; AME: 12.09; NEO: 14.27; NIV: 16.05	
**Cider**				30	AOH: 3	AOH: 21.56	
**Cereals, Legumes, Nuts**	Nigeria	LC-MS/MS	BEA MON NIV STC	MAIZE: 142	63.4; 64.1; 10.6; 26.1	8.14; 72.1; 20.0; 1.44	[15]
				MILLET :1	MON: 100	MON: 282	

				RICE: 23	52.2; 30.4; 0; 47.8	0.56; 11.1; ND; 0.96	
				SORGHUM: 24	70.8; 91.7; 0; 12.5	1.15; 60.9; ND; 0.96	
				COWPEA: 7	STC: 14.3	STC: 0.49	
				PEANUT: 53	47.2; 3.8; 9.4; 39.6	0.65; 2.5; 2.69; 6.43	
**Ale Beer**	Brazil	LC-QTOF-MS	ENN		0	ND	[16]
**Cereal-Based Foods For Infants And Young Children.**	Germany	LC-MS/MS	AOH	SPELT-BASED CEREALS; 5	60	0.7 ± 0.2	[17]
				WHEAT-BASED CEREALS: 5	40	0.7 ± 0.2	
				OAT-BASED CEREALS:6	67	0.6 ± 0.1	
				MILLET-BASED CEREALS: 3	100	0.6 ± 0.1	
				RICE-BASED CEREALS: 6	33	1±0.2	
				MIXED-GRAIN CEREALS: 13	85	0.9 ± 0.6	
**Tomato Purees And Juices, Sunflowers Seeds, Cereals And Derived Products**	Italy	LC-MS/MS	AOH TEA AME TEN	VEGETABLES AND VEGETABLE PRODUCTS: 37	0; 79; 0; 0	ND; 86.1; ND; ND	[18]
				GRAINS AND GRAIN-BASED PRODUCTS: 33	3; 58; 3; 6	14.3; 91.3; 6.3; 6.35	
				LEGUMES, NUTS AND OILSEEDS: 27	18; 100; 26; 44	31.5; 1451; 22.7; 154	
**Breast Milk**	Nigeria	LC-MS/MS	BEA	75	56	<LOQ-0.019 NG/ML	[19]

			ENN		ENNB: 9	<LOQ	
			NIV STC		0	ND	
**Oat Beverages, Almond Beverages, Rice Beverages, Soy Beverages**	Spain	LC-MS/MS	BEA	56	16	1.60 ± 04 NG/ML	[20]
			ENN		ENA: 3; ENA1:11; ENB: 22; ENB1:19	ENA: 0.59 ± 1.2 NG/ML; ENA1: 2.17 ± 2 NG/ML; ENB: 13.33 ± 68 NG/ML; ENB1: 4.46 ± 39 NG/ML	
			AME AOH TEN		AME:0; AOH: 3; TEN: 4	AME: ND; AOH: 0.21 ± 0.8 NG/ML; TEN: 21.30 ± 54 NG/ML	
**Gluten Free Pasta**	Italy	UHPLC-Q-EXACTIVE ORBITRAP MS	NIV	84	33.3	241.3	[21]
			BEA		9.5	19.6	
			ENN		ENA1: 4.8	1.7	
			FX NEO		0	ND	
**Infant Cereals**	China	UPLC–MS/MS	AME AOH TEA ENN	133	0	ND	[22]
**Infant Noodles**				229	ENA: 0; ENA1: 1.7; ENB: 9.6; ENB1: 9.2; AME: 4.8; AOH: 7.9: TEA: 86.5	ENA: ND; ENA1: 0.2; ENB:0.5; ENB1: 0.6; AME: 0.2; AOH: 0.3: TEA: 26.7	
**Infant Crackers**				277	ENA: 41.2; ENA1: 10.1; ENB:5.4; ENB1: 4; AME: 7.9; AOH: 3.2: TEA: 41.5	ENA: 2.2; ENA1: 0.4; ENB: 0.2; ENB1: 0.2; AME: 0.3; AOH: 0.2: TEA: 14.4	

**Rice**				58	ENA: 0; ENA1: 0; ENB: 0; ENB1: 34; AME: 0; AOH: 0 TEA: 0	ENA: ND; ENA1: ND; ENB: ND; ENB1: 4.7; AME: ND; AOH: ND TEA: ND	
**Millets**				69	ENNS: 0; AME: 4.3; AOH: 15.9; TEA: 78.3	ENA: ND; ENA1: ND; ENB: ND; ENB1: ND; AME: 0.2; AOH: 0.4 TEA: 73.6	
**Wheat Flour**				54	ENA: 0; ENA1: 0; ENB: 3.7; ENB1: 3.7; AME: 0; AOH: 16.7 TEA: 3.7	ENA: ND; ENA1: ND; ENB: 0.6; ENB1: 0.3; AME: ND; AOH: 3.3 TEA: 0.8	
**Unprocessed Wheat**	China	HPLC-MS/MS	NIV	338	45.3	266.4	[23]
**Corn Flour**	Egypt	UPLC-PDA	NIV	54	83.3	114	[24]
**Wheat Flour**				50	56	51	
**Agricultural Products And Processed Foods**	Korea	LC-MS/MS	STC	1135	4.1	0.08–10.07 NG/G	[25]
**Cereals, Herbs, Cereal-Based Food Products, Fruits, Oilseed Rape, Cocoa, Peanuts**	Poland	HPLC-MS/ MS	DAS FX NIV	92	0	ND	[26]
**Cereal-Based Food Products For Infants**	China	HPLC-MS/MS	AME AOH TEA	872	5.7	0.23	[27]
					7.5	0.66	
					47.5	18.26	

**And Young Children**							
**Rice Wine**	China	UPLC−HRMS/MS	STC		COMPARED WITH RICE, THE LEVELS OF STG IN SEPARATED FERMENTED WINE WAS SIGNIFICANTLY DECREASED BY 88.6%		[28]
**Cereals, Legume Potatoes Eggs Aquatic Foods Dairy Products Vegetables Fruits Sugar Beverages And Water Alcohol Beverages**	China	UHPLC-MS/MS	NIV	72	1.4	4.48	[29]
			FX		1.4	6.29	
			BEA		45.8		
			ENN		ENA: 23.6; ENA1:19.4; ENB: 58.3; ENB1: 31.9		
			NEO DAS MON		0		
			STC		29.2		
			AME AOH TEA TEN		AME: 27.8; AOH: 0; TEA:23.6; TEN: 30.6		
**Oat Products**	Brazil	HPLC–PDA	NIV	30	56.3	35.7-3571.4	[30]
**Milling Oat**	Eu	LC–MS/MS	NIV	281	(>LOQ) 91	94	[31]
			DAS	281	3.2	2.7	
			STC	177	2.3	0.6	
			FX	281	0	ND	
**Spring Barley**	Sweden	LC-MS/MS	NIV	177	66	44	[32]
**Spring Oats**				164	71	65	
**Winter Triticale**				144	24	9	
**Spring Wheat**				189	35	15	
**Winter Wheat**				741	43	18	
**Cereal-Based Baby Food**	Turkey	LC-MS/MS	STC	85	34.1	0.063 ± 0.018	[33]
**Corn**	China	CICLEIA	AOH	2	100		[34]
**Rice**				2	0		
**Flour**				2	100		

**Apple Juice**				2	50		
**Grape Juice**				2	50		
**Breakfast Cereals And Infant Cereals**	Brazil	HPLC-LC/MS/MS	NIV	31	0	ND	[35]
			FX	31			
			DAS	58			
**Paprika**	Spain	UHPLC-HRMS	ENN	26	ENB1: 3.8	12.0 ± 0.6	[36]
			BEA		0	ND	
**Oat**	Ireland	UHPLC-MS/MS	NIV	71	2.8		[37]
			STC		1.4		
			ENN		ENB: 19.7; ENB1: 8.5		
			BEA		1.4		
			FX NEO DAS AME AOH TEN		ND		
**Water**	China	UPLC-MS/MS	AME	289	0	ND	[38]
			AOH		0	ND	
			TEA		6.6	0.578 NG/L	
			TEN		3.1	0.509	
**Vegetable Oil**	China	SPE–HILIC–MS/MS	MON	224	0	ND	[39]
**Grain**	Germany	LC-MS/MS	AME AOH TEA TEN	28	AME: 14.2; AOH: 10.7; TEA: 7.1; TEN: 21.4	AME: 0.26-0.8; AOH: 0.97-1.8; TEA: 204-674; TEN: 0.66-9.2	[40]
			NEO		3.6	1.4	
			NIV		3.6	15	
			STC		3.6	4.4	
			DAS FX		0	ND	
**Cheese Balls**	Nigeria	LC-MS/MS	AME AOH TEA TEN ENN BEA MON NIV STC	10	MON:10; TEA: 10	MON: 32.6; TEA: 32.5	[41]
**Garri**				23	AME: 17.4	0.4	
**Granola**				18	AOH: 11.1; AME: 88.9; ENNS: 100; MON: 94.4; TEA: 72.2	AOH: 4.55; AME: 0.48; ENNS: 0.13-31.5; MON:	

						12.1; TEA: 19.4	
**Popcorn**				19	AME: 26.3; BEA: 5.3; ENB: 100; ENB1: 100; MON: 21.1; NIV: 21.1	AME: 0.74; BEA: 7.93; ENB: 0.15; ENB1: 0.27; MON: 32.4; NIV: 78.2	
**Rice**				58	ENA: 3.4; ENA1: 0; ENB: 34.5; ENB1: 22.4; BEA: 69	ENA: 1.04; ENA1: ND; ENB: 15.4; ENB1: 9.25; BEA: 37.2	
**Wheat**				53	ENA: 17; ENA1: 3.8; ENB: 30.2; ENB1: 39.6; BEA: 69.8	ENA: 1.83; ENA1: 0.33; ENB: 12.4; ENB1: 24.3; BEA: 58.4	
**Corn**	China	UPLC-MS/MS	ENN BEA	78	ENA: 78.2; ENA1: 19.2; ENB: 23.1; ENB1: 70.5; BEA: 35.9	ENA: 28.1; ENA1: 8.48; ENB: 11.9; ENB1: 45.8; BEA: 62.8	[42]
**Wheat Flours**				95	ENA: 20.0; ENA1: 6.3; ENB: 43.2; ENB1: 48.4; BEA: 70.5	ENA: 0.81; ENA1: 0.78; ENB: 5.49; ENB1: 5.66; BEA: 9.04	
**Corn Flours**				60	ENA: 28.3; ENA1: 6.7; ENB: 8.3; ENB1: 20.0; BEA: 3.3	ENA: 10.4; ENA1: 0.38; ENB: 4.31; ENB1: 10.8; BEA: 8.07	
**Green Coffee And Other Food Items**	Switzerland	LC-MS/MS	AME AOH TEA TEN	COFFEE: 78	AME: 1.3; AOH: 5.1; TEA: 2.6; TEN: 1.3		[43]
				OTHER FOODS: 138	AME: 21.0; AOH: 32.6; TEA: 63.0; TEN: 29.7		

**Kankankan: A Popular Spice**	Côte D'ivoire	UHPLC-MS/MS	BEA	75	28	<LOQ-134.2	[44]
**Ogi (A Fermented Cereal Beverage)**	Nigeria	ESI- LC-MS/MS	BEA	3	66.7	1.43	[45]
			MON		0	ND	
			STC		66.7	0.53	
**Barley**	Algeria	UHPLC-MS/MS	FX STC BEA ENN	30	FX: 10	190	[46]
**Maize**				30	FX: 80; BEA: 83.3; ENA1: 10; ENB1:6	FX: 281; BEA: 3.8; ENA1: 56.4; ENB1:60.9	
**Rice**				30	0	ND	
**Wheat**				30	FX: 10; BEA: 70; ENA: 23.3; ENA1: 46.7; ENB: 60; ENB1: 70	FX: 152; BEA: 155.4; ENA: 28.3; ENA1: 107; ENB: 1668; ENB1: 469	
**Norwegian Atlantic Salmon**	Spain	LC-Q-TOF-MS	ENN	10	0	ND	[47]
**Grain Cereals**	Latvia	NANOLC-ORBITRAP MS	NIV FX NEO ENN AOH AME TEN	110	NIV: 83; FX: 14; ENNS: 94; ALTS: 94	NIV: 71-4,780; FX: <50; ENNS: 3.5–2,073; ALTS: 0.72–307	[48]
**Pulses**				23	ENNS: 13; ALTS: 39	ENNS: 4.4–17; ALTS: 0.69–10	
**Oat Flake**	Czech Republic	U-HPLC-MS/MS	AOH AME TEN	20	AOH: 25; AME: 10; TEN: 20	AOH: 2; AME: 1.5; TEN: 1.75	[49]
			ENN		ENA: 30; ENA1: 75; ENB: 100; ENB1: 100	ENA: 2; ENA1: 4; ENB: 36; ENB1: 10	
			BEA		100	4	

**Cereal-Based Infant Foods**	Austria And Czech Republic	LC-MS/MS	STC NIV BEA ENN AME TEN TEA	35	STC: 23; NIV: 6; BEA: 14; ENA: 3; ENA1: 11; ENB: 60; ENB1:26; AME: 20; TEA: 31; TEN: 34	STC: 0.24; NIV: -; BEA: 1.9; ENA: -; ENA1: 0.7; ENB: 5.9; ENB1: 3.9; AME: 0.6; TEA: 48; TEN: 0.9	[50]
**Infant Formulae**				9	STC: 22; NIV: 22; ENB1:11; AME: 22	----------	
**Raw Flour**				15	BEA: 33; ENB: 13; ENB1:6; AME: 13; TEA: 40; TEN: 6	BEA: 1.4; ENB: -; ENB1; -; AME: -; TEA: 37; TEN: -	
**Edible Oil**	India	HPLC-FLD	AME	100	34	89.37	[51]
			AOH		35	251.5	
**Alcoholic Beverages, Baby Foods, Cereals And Cereal-Based, Legumes And Legume-Based Foods, Noodles, Snacks**	Korea	HPLC–UV	NIV	506	12	77.1	[52]
**Cereal Flour Products**	Korea	UHPLC-MS/MS	NIV	67	86.5	41.47	[53]
			FX		61.2	11.76	
**Maize**	South Africa	LC-MS/MS	AME TEA STC	24	STC: 4.2; TEA: 62.5	STC: 0.3; TEA: 3.1	[54]
**Maize Porridge**				20	STC: 10; TEA: 85	STC: 0.55; TEA: 1.25	
**Sorghum**				8	TEA: 100	TEA: 84.8	
**Wheat**				3	STC: 33.3; TEA: 100	STC: 0.4; TEA:1.8	

**Flour And Bread**	Poland	LC-TOF-HRMS	NIV	THE CONCENTRATIONS OF NIV IN THE FLOUR WERE 330 ± 21 MG/KG. THE CONCENTRATIONS OF NIV DID NOT CHANGE MUCH DURING MALT BREAD PRODUCTION.			[55]
**Cracker**	Brazil	UPLC-MS/MS	STC	60	0	ND	[56]
**Fruit Juice**	China	UHPLC-MS/MS	AME AOH TEA TEN	22	AME, TEN:0; TEA: 81.8; AOH: 13.6	AME, TEN: ND; TEA: 4.26 NG/ML; AOH:0.67 NG/ML	[57]
**Beer**	Poland	HPLC-MS/MS	NIV	69	1.4	<LOQ	[58]
			DAS		0	ND	
**Beer**	Poland	HPLC-UV	NIV	100	56	2.4±1.9 MG/L	[59]
**Cereals And Cereal Products, Legume and Related Products, Potatoes And Potato Products, Eggs And Egg Products, Aquatic Foods And Aquatic Food Products, Milk And Dairy Products, Vegetables And Vegetable Products, Fruits And Fruit Products,**	China	UPLC-MS/MS	AOH AME TEA TEN ENN BEA	60	AME: 31.7<br>TEA: 43.3<br>TEN: 28.3<br>AOH: 0<br>BEA: 33.3<br>ENA: 21.7<br>ENA1: 13.3<br>ENB: 43.3<br>ENB1: 28.3		[60]

**Sugar And Sugar Products, Beverages And Water, Alcohol Beverages**							
**Cereal Based Infant Food**	Germany	LC-MS/MS	AME AOH TEA TEN	19	AME: 89.4; AOH: 36.8; TEA: 5.3; TEN: 94.7	AME:0.24;[A] AOH:0.89; TEA: 50.2; TEN: 1.00	[61]
**Puree Infant Food**				6	AME: 83.3; AOH: 66.7; TEA: 66.7; TEN: 33.3	AME:1.53; AOH:9.62; TEA: 86.5; TEN: 0.23	
**Plant-Based Milk**	Spain	UHPLC-MS/MS	BEA ENN	32	BEA: 25	1.4	[62]
					ENA: 25	1.9	
					ENA1: 8.3	0.25	
					ENB: 25	13.5	
					ENB1: 25	13.4	
**Corn And Corn-Based**	China	HPLC-MS/MS	BEA ENN	158	BEA: 82.3; ENA: 55.1; ENA1: 8.2; ENB: 3.8; ENB1: 56.3	BEA: 65.26; ENA: 0.28; ENA1: 0.62; ENB: 1.19; ENB1: 0.13	[63]
**Wheat And Wheat-Based**				291	BEA: 56.4; ENA: 37.5; ENA1: 22; ENB: 58.1; ENB1: 54.6	BEA: 0.41; ENA: 0.19; ENA1: 0.96; ENB: 1.19; ENB1: 1.37	
**Cereal**	Korea	UPLC-MS/MS	BEA ENN AME AOH TEN	61	AOH: 24.6; AME: 18.0; TEN: 24.6; BEA: 6.6; ENA1: 3.3; ENB: 27.9; ENB1: 3.3	AOH: 3.52; AME: 15.61; TEN: 5.48; BEA: 15.05; ENA1: <LOQ; ENB: 2.73; ENB1: <LOQ	[64]

**Cereal-Based Product**				36	AOH: 55.6; AME: 27.8; TEN: 61.1; BEA: 11.1; ENB: 25;	AOH: 1.43; AME: 1.98; TEN: 16.42; BEA: 19.09; ENB: 1.45	
**Wheat**	Argentina	HPLC-DAD	AOH AME TEA	21	AOH: 23.8; AME: 38.1; TEA: 57.1	AOH: 11.63; AME: 9.4; TEA: 19190	[65]
**Bran**				21	TEA: 66.7	16760	
**Flour**				23	TEA:34.8	7360	
**Fresh Cheery**	China	LC-MS/MS	AME AOH TEA TEN	55	TEA: 66; AOH: 5.5; AME: 16; TEN: 36	TEA: 15.83; AOH: 0.87; AME: 0.031; TEN: 0.15	[66]
**Cheery Jam**				13	TEA: 100; AOH: 100; AME: 62; TEN: 100	TEA: 6.01; AOH: 0.67; AME: 0.016; TEN: 0.02	
**Dried Cheery**				12	TEA: 92; AOH: 92; AME: 92; TEN: 67	TEA: 54.22; AOH: 2.06; AME: 1.73; TEN: 0.11	
**Canned Cheery**				3	TEA: 100; AOH: 67; AME: 100; TEN: 100	TEA: 1.57; AOH: 0.07; AME: 0.004; TEN: 0.05	
**Soy-Based Burger**	Italy	UHPLC-Q-ORBITRAP HRMS	NIV NEO DAS ENN AOH AME	19	NIV, NEO, DAS: 0; AOH: 5.3; AME: 94.7; ENA: 31.6; ENA1: 36.8; ENB: 84.2; ENB1: 63.2	NIV, NEO, DAS: ND; AOH: 184.8; AME: 207.5; ENA: 323.8; ENA1: 67.3; ENB: 138.4; ENB1: 121.2	[67]

**Pasta**	Morocco	LC-MS/MS: ENN BEA; GC-MS/MS: NIV FX	ENN BEA NIV FX	106	BEA, NIV, FX: ND ENNA1: 9.4 ENNB: 67.9 ENNB1: 67.9		[68]
**Cereals**	Spain	LC-MS/MS AND GC-MS/MS	ENN BEA STC DAS NIV FX NEO	88	NEO: 45; NIV: 18; ENB: 2.72; ENNA1: 6; BEA: 7	NEO: 0.89;[A] NIV: 0.57; ENB: 39; ENNA1: 0.16; BEA: 0.25	[69]
**Legumes**				24	NEO: 17	NEO: 1.43	
**Vegetables**				141	NEO: 4; ENB: 3; ENNA1: 2; BEA: 9	NEO: 0.52; ENB: 0.03; ENNA1: 0.03; BEA: 0.87	
**Fish And Aquatic Products**				53	NEO: 4; ENA: 4; DAS: 4	NEO: 0.21; ENA: 0.23; DAS: 0.14	
				22	NIV: 18; ENB: 9; BEA: 5	NIV: 1.53; ENB: 0.1; BEA: 6.21	
**Family Cereal**	Nigeria	LC-MS/MS	AOH TEA BEA NIV MON	26	AOH: 15.4; BEA: 69.2; MON: 92.3	AOH: 0.6; BEA: 0.2; MON: 9.8	[70]
**Peanut Butter**				5	BEA: 80.0; MON: 60.0	BEA: 1.7; MON: 2.8	
**Ogi**				23	AOH: 4.3; BEA: 82.6; MON:17.4	AOH: 0.4; BEA: 1.3 MON:11.2	
**Tom Bran**				30	AOH: 30; BEA: 96.7; MON: 96.7; NIV: 10; TEA: 13.3	AOH: 2.1; BEA: 4.4; MON: 165; NIV: 16.4; TEA: 114	
**Milk**				36	BEA: 27.8	BEA: 0.2±0.1	
**Infant Formula**				17	AOH: 5.9; BEA: 17.6; MON: 11.8; NIV: 11.8	AOH: 0.7; BEA: 4.7;	

						MON: 13; NIV: 20.5	
**Wheat-Based Products**	Romania	GC-QQQ-MS/MS	FX DAS NIV NEO	181	0	ND	[71]
**Durum Wheat**	Italy	LC-HRMS	ENN BEA	16	ENA!1: 25; ENB: 93.8; ENB1: 87.5; BEA: 81.3	ENA!1: 3.5; ENB: 191.1; ENB1: 23.4; BEA: 4.2	[72]
**Tomato Sauces**	Austria	LC-MS/MS	AME AOH TEA TEN	12	AOH: 58.3; AME: 41.7; TEA: 83.3; TEN: 41.7		[73]
**Sunflower Seed Oils**				7	AOH: 42.9; AME: 71.4; TEA: 57.1; TEN: 100		
**Wheat Flour**				9	AOH: 11.1; AME: 0; TEA: 0; TEN: 11.1		
**Ready-To-Eat Food**	Spain	LC-MS/MS: ENNs BEA STC; GC-MS/MS: NIV NEO DAS FX	ENN BEA STC DAS NIV FX NEO	25	ENB: 20	26.91	[74]
					BEA, STC, DAS, NIV, FX, NEO: 0	ND	
**Vegetable**	China	UPLC-MS/MS	NEO DAS ENN BEA	SWEET PEPPER: 10	BEA: 50; ENA: 10	BEA: 15-57; ENA: 4.1	[75]
				CUCUMBER: 3	0	ND	
				TOMATO: 2	0	ND	
				POTATO: 5	0	ND	
**Liquorice**	China	UHPLC-MS/MS	AOH	31	45.2	<LOQ-520.6	[76]
			DAS STC		0	ND	
**Umqombothi**	South Africa	LC-MS/MS	NIV DAS	32	0	ND	[77]
			NEO		3	21 (MG/L)	
			FX		6	167	
			AOH		69	47	
			AME		34	41	
			STC		66	18	
			ENN		ENB: 75	17	
**Rice**	Pakistan		ENN STC	180	0	ND[A]	[78]

		GC-MS/MS: DAS NIV NEO; LC–MS/MS: STC BEA ENN	AME AOH FX NEO				
			NIV		28	13.8	
			DAS		23	1.6	
**Wheat Grain**	Brazil	LC-MS/MS	NIV	20	5	187.5	[79]
**Composite Food**	Sub-Saharan Africa	LC-MS/MS	STC	194	14.9	0.3	[80]
			DAS		0	ND	
**Cereal- Based Food**	Nigeria	LC-MS/MS	STC BEA ENN MON AME TEN	67	AME: 16; BEA: 66; ENA: 4; ENA1; 10; ENB: 43; ENB1: 25; MON: 9; STC: 12; TEN: 6	AME: 1.49; BEA: 0.29; ENA: 0.24; ENA1; 0.47; ENB: 0.91; ENB1: 0.50; MON: 3.14; STC: 0.29; TEN: 1.63	[81]
**Legume-Based Food**				2	AME: 100; BEA: 50; STC: 50	AME: 9.41; BEA: 0.33; STC: 0.13	
**Mixed Cereal- And Nut- Based Food**				13	AME: 8;; BEA: 100; ENA1: 15; ENB: 38; ENB1: 23; MON: 62; STC: 15; TEN: 8	AME: 0.26;; BEA: 0.3; ENA1: 0.26; ENB: 0.46; ENB1: 0.3; MON: 8.22; STC: 0.85; TEN: 0.4	
**Tuber-Based Food**				2	AME: 50;	AME: 0.26;	
**Infant Formulae**				4	0	ND	
**Egg Yolk**				3	0	ND	
**Okra Soup**				1	0	ND	
**Breast Milk**	Nigeria	LC-MS/MS	AME ENN BEA STC MON	22	AME: 95.5; BEA: 100; ENB: 72.7; ENB1: 22.7; STC: 4.6	AME: 3.0(NG/L); BEA: 3.0; ENB: 5.0; ENB1: 0.6; STC: 1.2	[82]

**Complementary Foods For Infant**				42	BEA: 78.6; ENA1: 16.7; ENB: 19; ENB1: 14.3; ENB2: 7.1; MON: 7.1	BEA: 3.8 NG/L; ENA1: 1.3; ENB: 2.1; ENB1: 4.1; ENB2: 0.22; MON: 8.6	
**Wheat Destined For Human Consumption**	Algeria	CE–QTOF MS/MS	ENN BEA	29	BEA: 17.2	13.0	[83]
					ENA: 27.5	25.2	
					ENA1: 20.6	32.0	
					ENB: 93.1	326	
					ENB1: 82.7	105	
**Wheat Flours**	China	LC-MS/MS	FX	299	11.7	8.5	[84]
			NIV		13.7	6.6	
			AOH		18.4	6.6	
			TEN		55.2	0.5	
			TEA		73.2	23.1	
			NEO		2.3	0.6	
**Edible Oil**	China	HPLC-ORBITRAP HRMS	STC DAS	13	STC: 15.4 DAS: 0	STC: 2.1-2.9 DAS: ND	[85]
**Soy Sauce And Bean Sauce**				11	0	ND	
**Vegetable Oil**	Thailand	LC–MS/MS	BEA	300	6.7	0.23-0.92	[86]
**Tomato-Based Products And Fruit-Based Products**	Italy	LC-ESI-MS/MS	AME AOH TEA TEN	57	AOH, AME, TEN: 0 TEA: 17.5	AOH, AME, TEN: ND TEA: <LOQ-814	[87]
**Raw Milk**	Portugal	UHPLC-MS/ MS	ALT: AOH AME DAS NEO STC FX	31	0	ND	[88]
			ENN		ENNS: 67.7		
			BEA		BEA: 90.3	0.09-2.96 NG/ML	
**Oat Grains**	Canada	UHPLC-HRMS	NIV BEA DAS ENN	2016: 43	NIV: 100; BEA: 90; DAS: 15; ENNS: 43	NIV: 252±27;[A] BEA: 25±4;	[89]

						DAS: 8±1.1; ENNS: 65±6	
				2017:60	NIV: 78; BEA: 80; DAS: 7; ENNS: 39	NIV: 234±16; BEA: 41±4; DAS: 7±0.6; ENNS: 16±2	
				2018: 65	NIV: 98; BEA: 71; DAS: 5; ENNS: 17	NIV: 145±12; BEA: 24±5; DAS: 6±0.5; ENNS: 10±1	
**Plant-Based Protein,**	Switzerland	UHPLC-HRMS	AOH AME TEA TEN BEA DAS ENN NEO NIV STC FX	6	BEA: 16.7; ENA: 0; ENA1: 0; ENB: 33.3; ENB1: 16.7; TEN: 16.7; TEA: 0	IDENTIFIED BY THE SCREENING METHOD	[90]
**Cereals, And Pseudo-Cereal**				6	BEA: 0; ENA: 16.7; ENA1: 33.3; ENB: 33.3; ENB1: 50; TEN: 0; TEA: 33.3	IDENTIFIED BY THE SCREENING METHOD	
**Barley**	Czech Republic	UPLC/MS/MS	ENN BEA NIV	12	NIV: 58; BEA: 66.7; ENA: 25; ENA1: 75; ENB: 100; ENB1: 92	NIV: 239; BEA: 57; ENA: 5; ENA1: 32; ENB: 226; ENB1: 105	[91]
**Oats**				12	NIV: 17; BEA: 33; ENA: 0; ENA1:0; ENB: 33; ENB1: 17	NIV: 68; BEA: 10; ENA: ND; ENA1: ND; ENB: 10; ENB1: 4	
**Barley**	Argentina	HPLC-MS/MS	AME AOH TEA	2014: 26	TEA: 11.5; AOH: 80.8	TEA: 1646; AOH: 623	[92]
				2015: 34	TEA; 55.9; AOH: 47.1; AME: 14.7	TEA; 1397; AOH: 801; AME: 2201	
**Small Grain Cereals**	France	LC/MS- MS	ENN	1240	74.75		[93]

**Maize**	Serbia	LC-MS/MS	BEA MON ENN	190	BEA: 60; MON: 73.7; ENA: 3.2; ENA1: 4.1; ENB: 1.6; ENB1: 2.1	BEA: 11.05; MON: 364.57; ENA: 3.13; ENA1:; 3.72; ENB: 3.05; ENB1: 5.41	[94]
**Fruits, Vegetables, And Their Derivatives**	China	*UHPLC–MS/MS*	ALT: AOH AME TEA TEN	270	AOH: 14.1; AME: 4.4; TEA: 22.6; TEN: 3.3	AOH: 4.3; AME: 3.4; TEA: 48.5; TEN: 5.4	[95]
**Sorghum**	Ethiopia	LC-MS/MS	NIV DAS MON BEA ENNS STC TEA AOH AME TEN	80	NIV: 16; DAS: 16; MON: 93; BEA: 21; ENA1: 2.5; ENB: 10; ENB1: 3.75; STC: 30; TEA: 93; AOH: 59; AME: 50; TEN: 58	NIV: 1.66;[A] DAS: 0.88; MON: 58.0; BEA: 0.57; ENA1: 0.02; ENB: 0.02; ENB1: 0.03; STC: 2.80; TEA: 155; AOH: 3.39; AME: 2.38; TEN: 1.07	[96]
**Cheese**	Spain	UHPLC-MS/MS	AOH AME BEA DAS ENNS NEO STC	38	AOH, DAS, NEO: 0; AME: 28.9; BEA: 100; ENA: 31.6; ENA1: 57.9; ENB: 100; ENB1: 76.3; STC: 34.2	AOH, DAS, NEO: ND; AME: 2.38; BEA: 3.94; ENA: 1.22; ENA1: 1.67; ENB: 1.86; ENB1: 1.76; STC: 1.90	[97]
**Aromatic And Medicinal Plants**	Morocco	Q-TOF-LC/MS	AOH TEN ENNS BEA	40	ENA1: 2.5; ENB: 10; AOH: 85; TEN: 17.5; BEA: 0	ENA1: 0.16 NG/G; ENB: 0.05; AOH: 126.2; TEN: 1.47; BEA: ND	[98]
**Breast Milk**	Nigeria	LC-MS/MS	AOH AME TEN	4	AME: 100; BEA: 100; ENA: 25;	AME: 9.1 NG/L; BEA:	[99]

			BEA ENNS NIV STC		ENA1: 25; ENB: 100; ENB1: 100; AOH, TEN, NIV, STC: 0	8.75; ENA: 0.5; ENA1: 0.9; ENB: 1.88; ENB1: 0.5; AOH, TEN, NIV, STC: ND	
**Maize**	Togo	HPLC-MS/MS	NIV NEO FX DAS STC ALT: AOH	55	DAS: 17; STC: 2; NIV, NEO, FX, AOH: 0	DAS: 2.6; STC: 10; NIV, NEO, FX, AOH: ND	[100]
**Sorghum**				12	NIV: 17; DAS: 42; STC: 50; NEO, FX, AOH: 0	NIV: 49.5; DAS: 4.8; STC: 18.5; NEO, FX, AOH: ND	
**Rye**	Poland	LC-MS	NIV DAS	60	NIV: 10; DAS: 0	NIV: 9.96; DAS:ND	[101]
**Tea**	China	HPLC-MS/MS	STC	126	13.5	0.13-4.48	[102]
**Cereal And Cereal Based Products**	Japan	LC-MS/MS	STC	451	25.3	0.54	[103]
**Azuki Bean**				39	5.1	0.08	
**Wine**				63	0	ND	
**Beer**				30	0	ND	
**Edible And Medicinal Herbs**	China	UPLC-MS/MS	AOH TEN AME TEA	260	AOH: 10.4	<LOQ-193.04	[104]
					TEN: 5	<LOQ-22.52	
					AME: 17.3	<LOQ-15.97	
					TEA: 8.1	28.62-2102.31	
**Grains and grain-based products**	Finland, Italy, the Netherlands, Norway, Sweden and the United Kingdom (UK)	LC-MS/MS	MON	1457		56.7	[105]
**Vegetables and vegetable products**				75		50.0	

**(including fungi)**							
**Starchy roots and tubers**				2		50.0	
**Legumes, nuts and oilseeds**				871		50.0	
**Fruit and fruit products**				453		50.0	
**Sugar and confectionary**				1		50.0	
**Animal and vegetable fats and oils**				39		50.0	
**Fruit and vegetable juices**				74		50.0	
**Alcoholic beverages**				198		50.0	
**Herbs, spices and condiments**				28		50.0	
**Food for infants and small children**				1	-	50.0	
**Products for special nutritional use**				1		50.0	
**Snacks, desserts, and other foods**				4		75.5	
**Tomato-based preparation**	Switzerland/Turkey/Germany	-	AOH; TeA	-	-	32; 372	[106]
**Strained tomatoes**	Italy/Germany	-	AOH; TeA	-	-	81.7; 549.1	[107]

ND = not detected; LC-MS/MS = Liquid-Chromatography with tandem Mass Spectrometry; UPLC = Ultra-Performance Liquid Chromatography; HPLC = High-Performance Liquid Chromatography; Q-TOF = Quadrupole Time of Flight; ESI = Electrospray Ionization; UV/VIS = Ultraviolet visible; HRMS = High-Resolution Mass Spectrometry; DAD = Diode-Array Detection; Alt = Alternaria; AOH = alternariol; AME = alternariol methyl ether; TEN = tentoxin; TeA = tenuazonic acid; BEA = Beauvericin; DAS = diacetoxyscirpenol; ENNs = enniatins; FX = Fusarenon-X; MON = moniliformin; NEO = neosolaniol; NIV = nivalenol; STC = sterigmatocystin

References

1. Ezekiel, C.N.; Ayeni, K.I.; Ezeokoli, O.T.; Sulyok, M.; Van Wyk, D.A.B.; Oyedele, O.A.; Akinyemi, O.M.; Chibuzor-Onyema, I.E.; Adeleke, R.A.; Nwangburuka, C.C.; et al. High-Throughput Sequence Analyses of Bacterial Communities and Multi-Mycotoxin Profiling during Processing of Different Formulations of Kunu, a Traditional Fermented Beverage. *Front. Microbiol.* **2019**, *10*, 1–17, doi:10.3389/fmicb.2018.03282.
2. Park, J.; Kim, D.H.; Moon, J.Y.; An, J.A.; Kim, Y.W.; Chung, S.H.; Lee, C. Distribution Analysis of Twelve Mycotoxins in Corn and Corn-Derived Products by LC-MS/MS to Evaluate the Carry-over Ratio during Wet-Milling. *Toxins (Basel).* **2018**, *10*, 1–15, doi:10.3390/toxins10080319.
3. Bryła, M.; Ksieniewicz-Woźniak, E.; Waśkiewicz, A.; Szymczyk, K.; Jędrzejczak, R. Natural Occurrence of Nivalenol, Deoxynivalenol, and Deoxynivalenol-3-Glucoside in Polish Winter Wheat. *Toxins (Basel).* **2018**, *10*, doi:10.3390/toxins10020081.
4. Nogueira, M.S.; Decundo, J.; Martinez, M.; Dieguez, S.N.; Moreyra, F.; Moreno, M.V.; Stenglein, S.A. Natural Contamination with Mycotoxins Produced by Fusarium Graminearum and Fusarium Poae in Malting Barley in Argentina. *Toxins (Basel).* **2018**, *10*, doi:10.3390/toxins10020078.
5. Rozentale, I.; Bogdanova, E.; Bartkevics, V. A Rapid and Sensitive Method for the Control of Selected Regulated and Emerging Mycotoxins in Beer. *World Mycotoxin J.* **2018**, *11*, 503–517, doi:10.3920/WMJ2017.2298.
6. Yoshinari, T.; Takeda, N.; Watanabe, M.; Sugita-Konishi, Y. Development of an Analytical Method for Simultaneous Determination of the Modified Forms of 4,15-Diacetoxyscirpenol and Their Occurrence in Japanese Retail Food. *Toxins (Basel).* **2018**, *10*, doi:10.3390/toxins10050178.
7. Chung, S.W.C.; Wu, A.H.T. Development and Validation of an Analytical Method for the Analysis of Sterigmatocystin in Roasted Coffee Beans and Black Pepper Using Liquid Chromatography-Tandem Mass Spectrometry. *Food Addit. Contam. - Part A Chem. Anal. Control. Expo. Risk Assess.* **2020**, *37*, 355–362, doi:10.1080/19440049.2019.1693635.
8. Liu, B.; Ni, M.; Shan, X.; Xie, J.; Dai, Y.; Zhang, C. Simulatenous determination of beauvericin and four enniatins in eggs by ultra-performance liquid chromatography-tandem mass spectrometry coupled with cold-induced liquid-liquid extraction and dispersive solid phase extraction. *Chin. J. Chromatogr.*

2021.

9. Liang, H.; Hou, Q.; Zhou, Y.; Zhang, L.; Yang, M.; Zhao, X. Centrifugation-Assisted Solid-Phase Extraction Coupled with UPLC-MS/MS for the Determination of Mycotoxins in ARECAE Semen and Its Processed Products. *Toxins (Basel).* **2022**, *14*, doi:10.3390/toxins14110742.

10. Testempasis, S.I.; Kamou, N.N.; Papadakis, E.N.; Menkissoglu-Spiroudi, U.; Karaoglanidis, G.S. Conventional vs. Organic Vineyards: Black Aspergilli Population Structure, Mycotoxigenic Capacity and Mycotoxin Contamination Assessment in Wines, Using a New Q-TOF MS-MS Detection Method. *Food Control* **2022**, *136*, 108860, doi:10.1016/j.foodcont.2022.108860.

11. Radić, B.; Janić Hajnal, E.; Mandić, A.; Krulj, J.; Stojanović, Z.; Kos, J. Development and validation of an HPLC–dad method for the determination of Moniliformin in maize. *Journal of Food Processing and Preservation* **2021**, *46*.

12. Guo, W.; Fan, K.; Nie, D.; Meng, J.; Huang, Q.; Yang, J.; Shen, Y.; Tangni, E.K.; Zhao, Z.; Wu, Y.; et al. Development of a QuEChERS-Based UHPLC-MS/MS Method for Simultaneous Determination of Six Alternaria Toxins in Grapes. *Toxins (Basel).* **2019**, *11*, 1–13, doi:10.3390/toxins11020087.

13. Scheibenzuber, S.; Dick, F.; Bretträger, M.; Gastl, M.; Asam, S.; Rychlik, M. Development of Analytical Methods to Study the Effect of Malting on Levels of Free and Modified Forms of Alternaria Mycotoxins in Barley. *Mycotoxin Res.* **2022**, *38*, 137–146, doi:10.1007/s12550-022-00455-1.

14. Carballo, D.; Fernández-Franzón, M.; Ferrer, E.; Pallarés, N.; Berrada, H. Dietary Exposure to Mycotoxins through Alcoholic and Non-Alcoholic Beverages in Valencia, Spain. *Toxins (Basel).* **2021**, *13*, 1–20, doi:10.3390/toxins13070438.

15. Ezekiel, C.N.; Ayeni, K.I.; Akinyemi, M.O.; Sulyok, M.; Oyedele, O.A.; Babalola, D.A.; Ogara, I.M.; Krska, R. Dietary Risk Assessment and Consumer Awareness of Mycotoxins among Household Consumers of Cereals, Nuts and Legumes in North-Central Nigeria. *Toxins (Basel).* **2021**, *13*, 1–23, doi:10.3390/toxins13090635.

16. Oliveira Lago, L.; Barreto Hoffmann Maciel, J.; Pires Costa, G.; Peixoto Mallmann, L.; Fonseca Veras, F.; Welke, J.E. Fate of Enniatins in the Ale Beer Production Stages Analyzed by a Validated Method Based on Matrix-Matched Calibration and LC-QToF-MS. *Food Chem.* **2022**, *384*,

doi:10.1016/j.foodchem.2022.132484.

17. Rehagel, C.; Akineden, Ö.; Usleber, E. Microbiological and Mycotoxicological Analyses of Processed Cereal-Based Complementary Foods for Infants and Young Children from the German Market. *J. Food Sci.* **2022**, *87*, 1810–1822, doi:10.1111/1750-3841.16106.

18. Lattanzio, V. M. T.; Verdini, E.; Sdogati, S.; Bibi, R.; Ciasca, B.; Pecorelli, I. Monitoring *alternaria* toxins in Italian food to support upcoming regulation. *Food Additives & Contaminants: Part B* **2021**, *15*, 42–51.

19. Braun, D.; Ezekiel, C.N.; Abia, W.A.; Wisgrill, L.; Degen, G.H.; Turner, P.C.; Marko, D.; Warth, B. Monitoring Early Life Mycotoxin Exposures via LC-MS/MS Breast Milk Analysis. *Anal. Chem.* **2018**, *90*, 14569–14577, doi:10.1021/acs.analchem.8b04576.

20. Juan, C.; Mañes, J.; Juan-García, A.; Moltó, J.C. Multimycotoxin Analysis in Oat, Rice, Almond and Soy Beverages by Liquid Chromatography-Tandem Mass Spectrometry. *Appl. Sci.* **2022**, *12*, doi:10.3390/app12083942.

21. Tolosa, J.; Rodríguez-Carrasco, Y.; Graziani, G.; Gaspari, A.; Ferrer, E.; Mañes, J.; Ritieni, A. Mycotoxin Occurrence and Risk Assessment in Gluten-Free Pasta through Uhplc-q-Exactive Orbitrap Ms. *Toxins (Basel).* **2021**, *13*, 1–13, doi:10.3390/toxins13050305.

22. Ji, X.; Xiao, Y.; Wang, W.; Lyu, W.; Wang, X.; Li, Y.; Deng, T.; Yang, H. Mycotoxins in Cereal-Based Infant Foods Marketed in China: Occurrence and Risk Assessment. *Food Control* **2022**, *138*, 108998, doi:10.1016/j.foodcont.2022.108998.

23. Zhao, J.; Cheng, T.; Xu, W.; Han, X.; Zhang, J.; Zhang, H.; Wang, C.; Fanning, S.; Li, F. Natural Co-Occurrence of Multi-Mycotoxins in Unprocessed Wheat Grains from China. *Food Control* **2021**, *130*, doi:10.1016/j.foodcont.2021.108321.

24. Gab-Allah, M.A.; Tahoun, I.F.; Yamani, R.N.; Rend, E.A.; Shehata, A.B. Natural Occurrence of Deoxynivalenol, Nivalenol and Deoxynivalenol-3-Glucoside in Cereal-Derived Products from Egypt. *Food Control* **2022**, *137*, 108974, doi:10.1016/j.foodcont.2022.108974.

25. Kang, Y. W.; Baek, S.-K.; Choi, M.; Lee, H. J.; Koo, Y. E. Occurrence and risk assessment of sterigmatocystin in agricultural products and processed foods in Korea. *Food Additives & Contaminants: Part A* **2022**, *39*, 373–381.

26. Kowalska, G.; Kowalski, R. Occurrence of Mycotoxins in Selected Agricultural and Commercial Products Available in Eastern Poland. *Open Chem.* **2021**, *19*, 653–664, doi:10.1515/chem-2021-0056.

27. Products, C.F.; Children, Y. Probabilistic Risk Assessment of Combined Exposure to Deoxynivalenol and Emerging Alternaria Toxins in in China. **2022**, 1–16.

28. Zhang, J.; Xu, L.; Xu, X.; Wu, X.; Kuang, H.; Xu, C. Profiles of Sterigmatocystin and Its Metabolites during Traditional Chinese Rice Wine Processing. *Biosensors* **2022**, *12*, 1–14, doi:10.3390/bios12040212.

29. Qiu, N.; Sun, D.; Zhou, S.; Li, J.; Zhao, Y.; Wu, Y. Rapid and Sensitive UHPLC-MS/MS Methods for Dietary Sample Analysis of 43 Mycotoxins in China Total Diet Study. *J. Adv. Res.* **2022**, *39*, 15–47, doi:10.1016/j.jare.2021.10.008.

30. Cerqueira, M.B.R.; de Borba, V.S.; Rodrigues, M.H.P.; Silveira, C.O.; Badiale-Furlong, E.; Kupski, L. Reliable and Accessible Method for Trichothecenes Type B Determination in Oat Products. *Food Anal. Methods* **2023**, *16*, 83–95, doi:10.1007/s12161-022-02397-x.

31. Meyer, J.C.; Hennies, I.; Wessels, D.; Schwarz, K. Survey of Mycotoxins in Milling Oats Dedicated for Food Purposes between 2013 and 2019 by LC–MS/MS. *Food Addit. Contam. - Part A Chem. Anal. Control. Expo. Risk Assess.* **2021**, *38*, 1934–1947, doi:10.1080/19440049.2021.1950931.

32. Karlsson, I.; Mellqvist, E.; Persson, P. Temporal and Spatial Dynamics of Fusarium Spp. and Mycotoxins in Swedish Cereals during 16 Years. *Mycotoxin Res.* **2023**, *39*, 3–18, doi:10.1007/s12550-022-00469-9.

33. Er Demirhan, B.; Demirhan, B. The Investigation of Mycotoxins and Enterobacteriaceae of Cereal-Based Baby Foods Marketed in Turkey. *Foods* **2021**, *10*, doi:10.3390/foods10123040.

34. Yao, C.Y.; Xu, Z.L.; Wang, H.; Zhu, F.; Luo, L.; Yang, J.Y.; Sun, Y.M.; Lei, H.T.; Tian, Y.X.; Shen, Y.D. High Affinity Antibody Based on a Rationally Designed Hapten and Development of a Chemiluminescence Enzyme Immunoassay for Quantification of Alternariol in Fruit Juice, Maize and Flour. *Food Chem.* **2019**, *283*, 359–366, doi:10.1016/j.foodchem.2018.12.127.

35. Mallmann, C.A.; Tyska, D.; Almeida, C.A.A.; Oliveira, M.S.; Gressler, L.T. Mycotoxicological Monitoring of Breakfast and Infant Cereals Marketed in Brazil. *Int. J. Food Microbiol.* **2020**, *331*, 108628, doi:10.1016/j.ijfoodmicro.2020.108628.

36. García-Nicolás, M.; Arroyo-Manzanares, N.; Campillo, N.; Viñas, P. Cellulose-Ferrite Nanocomposite for Monitoring Enniatins and Beauvericins in Paprika by Liquid Chromatography and High-Resolution Mass Spectrometry. *Talanta* **2021**, *226*, doi:10.1016/j.talanta.2021.122144.

37. De Colli, L.; Elliott, C.; Finnan, J.; Grant, J.; Arendt, E.K.; McCormick, S.P.; Danaher, M. Determination of 42 Mycotoxins in Oats Using a Mechanically Assisted QuEChERS Sample Preparation and UHPLC-MS/MS Detection. *J. Chromatogr. B Anal. Technol. Biomed. Life Sci.* **2020**, *1150*, 122187, doi:10.1016/j.jchromb.2020.122187.

38. Zhang, Y.; Li, H.; Zhang, J.; Shao, B. Determination of Alternaria Toxins in Drinking Water by Ultra-Performance Liquid Chromatography Tandem Mass Spectrometry. *Environ. Sci. Pollut. Res.* **2019**, *26*, 22485–22493, doi:10.1007/s11356-019-05483-6.

39. Chen, R.; Li, J.; Yang, Z.; Zhang, A.; Li, X.; Qi, P.; Li, J.; Zhang, J. Determination of Moniliformin in Vegetable Oil by Solid-Phase Extraction–Hydrophilic Interaction Chromatography–Tandem Mass Spectrometry. *Chromatographia* **2020**, *83*, 903–907, doi:10.1007/s10337-020-03901-3.

40. Rausch, A.K.; Brockmeyer, R.; Schwerdtle, T. Development and Validation of a QuEChERS-Based Liquid Chromatography Tandem Mass Spectrometry Multi-Method for the Determination of 38 Native and Modified Mycotoxins in Cereals. *J. Agric. Food Chem.* **2020**, *68*, 4657–4669, doi:10.1021/acs.jafc.9b07491.

41. Ezekiel, C.N.; Oyedele, O.A.; Kraak, B.; Ayeni, K.I.; Sulyok, M.; Houbraken, J.; Krska, R. Fungal Diversity and Mycotoxins in Low Moisture Content Ready-To-Eat Foods in Nigeria. *Front. Microbiol.* **2020**, *11*, doi:10.3389/fmicb.2020.00615.

42. Lin, X.; Zhang, Q.; Zhang, Y.; Li, J.; Zhang, M.; Hu, X.; Li, F. Further Data on the Levels of Emerging Fusarium Mycotoxins in Cereals Collected from Tianjin, China. *Food Addit. Contam. Part B Surveill.* **2021**, *14*, 74–80, doi:10.1080/19393210.2021.1873425.

43. Mujahid, C.; Savoy, M.C.; Baslé, Q.; Woo, P.M.; Ee, E.C.Y.; Mottier, P.; Bessaire, T. Levels of Alternaria Toxins in Selected Food Commodities Including Green Coffee. *Toxins (Basel).* **2020**, *12*, 1–17, doi:10.3390/toxins12090595.

44. Yapo, A.E.; Strub, C.; Durand, N.; Ahoua, A.R.C.; Schorr-Galindo, S.; Bonfoh, B.; Fontana, A.; Koussémon, M. Mass Spectrometry-Based Detection and Risk Assessment of Mycotoxin Contamination of ‘Kankankan’ Used for Roasted Meat Consumption in Abidjan, Côte d’Ivoire. *Food Addit. Contam. - Part A Chem. Anal. Control. Expo. Risk Assess.* **2020**, *37*, 1564–1578, doi:10.1080/19440049.2020.1784468.

45. Chibuzor-Onyema, I.E.; Ezeokoli, O.T.; Sulyok, M.; Notununu, I.; Petchkongkaew, A.; Elliott, C.T.; Adeleke, R.A.; Krska, R.; Ezekiel, C.N. Metataxonomic Analysis of Bacterial Communities and Mycotoxin Reduction during Processing of Three Millet Varieties into Ogi, a Fermented Cereal Beverage. *Food Res. Int.* **2021**, *143*, 110241, doi:10.1016/j.foodres.2021.110241.

46. Mahdjoubi, C.K.; Arroyo-manzanares, N.; Hamini-kadar, N. Multi-Mycotoxin Occurrence and Exposure. *Toxins (Basel).* **2020**, *12*, 1–18.

47. Tolosa, J.; Barba, F.J.; Pallarés, N.; Ferrer, E. Mycotoxin Identification and In Silico Toxicity Assessment Prediction in Atlantic Salmon. *Mar. Drugs* **2020**, *18*, doi:10.3390/md18120629.

48. Reinholds, I.; Jansons, M.; Fedorenko, D.; Pugajeva, I.; Zute, S.; Bartkiene, E.; Bartkevics, V. Mycotoxins in Cereals and Pulses Harvested in Latvia by NanoLC-Orbitrap MS. *Food Addit. Contam. Part B Surveill.* **2021**, *14*, 115–123, doi:10.1080/19393210.2021.1892204.

49. Dropa, T.; Dzuman, Z.; Jonatova, P. Mycotoxins in Oat Flakes – Changes during Production and Occurrence on the Czech Market. *Czech J. Food Sci.* **2021**, *39*, 131–139, doi:10.17221/247/2020-CJFS.

50. Braun, D.; Eiser, M.; Puntscher, H.; Marko, D.; Warth, B. Natural Contaminants in Infant Food: The Case of Regulated and Emerging Mycotoxins. *Food Control* **2021**, *123*, 107676, doi:10.1016/j.foodcont.2020.107676.

51. Bansal, M.; Saifi, I.J.; Dev, I.; Sonkar, A.K.; Dixit, S.; Singh, S.P.; Ansari, K.M. Occurrence of Alternariol and Alternariolmonomethyl Ether in Edible Oils: Their Thermal Stability and Intake Assessment in State of Uttar Pradesh, India. *J. Food Sci.* **2021**, *86*, 1124–1131, doi:10.1111/1750-3841.15629.

52. Lee, S.Y.; Woo, S.Y.; Tian, F.; Song, J.; Michlmayr, H.; Kim, J.B.; Chun, H.S. Occurrence of Deoxynivalenol, Nivalenol, and Their Glucosides in Korean Market Foods and Estimation of Their Population Exposure through Food Consumption. *Toxins (Basel).* **2020**, *12*, 1–12, doi:10.3390/toxins12020089.

53. Gab-Allah, M.A.; Mekete, K.G.; Choi, K.; Kim, B. Occurrence of Major Type-B Trichothecenes and Deoxynivalenol-3-Glucoside in Cereal-Based Products from Korea. *J. Food Compos. Anal.* **2021**, *99*, 103851, doi:10.1016/j.jfca.2021.103851.
54. Tebele, S.M.; Gbashi, S.; Adebo, O.; Changwa, R.; Naidu, K.; Njobeh, P.B. Quantification of Multi-Mycotoxin in Cereals (Maize, Maize Porridge, Sorghum and Wheat) from Limpopo Province of South Africa. *Food Addit. Contam. - Part A Chem. Anal. Control. Expo. Risk Assess.* **2020**, *37*, 1922–1938, doi:10.1080/19440049.2020.1808715.
55. Bryła, M.; Ksieniewicz-Wózniak, E.; Waśkiewicz, A.; Yoshinari, T.; Szymczyk, K.; Podolska, G.; Gwiazdowski, R.; Kubiak, K. Transformations of Selected Fusarium Toxins and Their Modified Forms during Malt Loaf Production. *Toxins (Basel).* **2020**, *12*, 1–15, doi:10.3390/toxins12060385.
56. Medina, B.G.; Sartori, A.V.; de MORAES, M.H.P.; Cardoso, M.H.W.M.; Jacob, S.D.C. Validation and Application of an Analytical Method for the Determination of Mycotoxins in Crackers by UPLC-MS/MS. *Food Sci. Technol.* **2019**, *39*, 583–591, doi:10.1590/fst.33717.
57. Guo, W.; Yang, J.; Niu, X.; Tangni, E.K.; Zhao, Z.; Han, Z. A Reliable and Accurate UHPLC-MS/MS Method for Screening of: Aspergillus, Penicillium and Alternaria Mycotoxins in Orange, Grape and Apple Juices. *Anal. Methods* **2021**, *13*, 192–201, doi:10.1039/d0ay01787f.
58. Grajewski, J.; Kosicki, R.; Twarużek, M.; Błajet-Kosicka, A. Occurrence and Risk Assessment of Mycotoxins through Polish Beer Consumption. *Toxins (Basel).* **2019**, *11*, 1–12, doi:10.3390/toxins11050254.
59. Bryła, M.; Ksieniewicz-Woźniak, E.; Waśkiewicz, A.; Szymczyk, K.; Jędrzejczak, R. Co-Occurrence of Nivalenol, Deoxynivalenol and Deoxynivalenol-3-Glucoside in Beer Samples. *Food Control* **2018**, *92*, 319–324, doi:10.1016/j.foodcont.2018.05.011.
60. Sun, D.; Qiu, N.; Zhou, S.; Lyu, B.; Zhang, S.; Li, J.; Zhao, Y.; Wu, Y. Development of Sensitive and Reliable UPLC-MS/MS Methods for Food Analysis of Emerging Mycotoxins in China Total Diet Study. *Toxins (Basel).* **2019**, *11*, 1–16, doi:10.3390/toxins11030166.
61. Gotthardt, M.; Asam, S.; Gunkel, K.; Moghaddam, A.F.; Baumann, E.; Kietz, R.; Rychlik, M. Quantitation of Six Alternaria Toxins in Infant Foods Applying Stable Isotope Labeled Standards. *Front. Microbiol.* **2019**, *10*, doi:10.3389/fmicb.2019.00109.

62. Arroyo-Manzanares, N.; Hamed, A.M.; García-Campaña, A.M.; Gámiz-Gracia, L. Plant-Based Milks: Unexplored Source of Emerging Mycotoxins. A Proposal for the Control of Enniatins and Beauvericin Using UHPLC-MS/MS. *Food Addit. Contam. Part B Surveill.* **2019**, *12*, 296–302, doi:10.1080/19393210.2019.1663276.

63. Han, X.; Xu, W.; Zhang, J.; Xu, J.; Li, F. Natural Occurrence of Beauvericin and Enniatins in Corn-and Wheat-Based Samples Harvested in 2017 Collected from Shandong Province, China. *Toxins (Basel).* **2019**, *11*, 1–12, doi:10.3390/toxins11010009.

64. Kim, D.B.; Song, N.E.; Nam, T.G.; Lee, S.; Seo, D.; Yoo, M. Occurrence of Emerging Mycotoxins in Cereals and Cereal-Based Products from the Korean Market Using LC-MS/MS. *Food Addit. Contam. - Part A Chem. Anal. Control. Expo. Risk Assess.* **2019**, *36*, 289–295, doi:10.1080/19440049.2018.1562233.

65. Romero Bernal, Á.R.; Reynoso, C.M.; García Londoño, V.A.; Broggi, L.E.; Resnik, S.L. Alternaria Toxins in Argentinean Wheat, Bran, and Flour. *Food Addit. Contam. Part B Surveill.* **2019**, *12*, 24–30, doi:10.1080/19393210.2018.1509900.

66. Qiao, X.; Yin, J.; Yang, Y.; Zhang, J.; Shao, B.; Li, H.; Chen, H. Determination of Alternaria Mycotoxins in Fresh Sweet Cherries and Cherry-Based Products: Method Validation and Occurrence. *J. Agric. Food Chem.* **2018**, *66*, 11846–11853, doi:10.1021/acs.jafc.8b05065.

67. Rodríguez-Carrasco, Y.; Castaldo, L.; Gaspari, A.; Graziani, G.; Ritieni, A. Development of an UHPLC-Q-Orbitrap HRMS Method for Simultaneous Determination of Mycotoxins and Isoflavones in Soy-Based Burgers. *LWT* **2019**, *99*, 34–42, doi:10.1016/j.lwt.2018.09.046.

68. Bouafifssa, Y.; Manyes, L.; Rahouti, M.; Mañes, J.; Berrada, H.; Zinedine, A.; Fernández-Franzón, M. Multi-Occurrence of Twenty Mycotoxinsin Pasta and a Risk Assessment in the Moroccan Population. *Toxins (Basel).* **2018**, *10*, 1–14, doi:10.3390/toxins10110432.

69. Carballo, D.; Moltó, J.C.; Berrada, H.; Ferrer, E. Presence of Mycotoxins in Ready-to-Eat Food and Subsequent Risk Assessment. *Food Chem. Toxicol.* **2018**, *121*, 558–565, doi:10.1016/j.fct.2018.09.054.

70. Ojuri, O.T.; Ezekiel, C.N.; Sulyok, M.; Ezeokoli, O.T.; Oyedele, O.A.; Ayeni, K.I.; Eskola, M.K.; Šarkanj, B.; Hajšlová, J.; Adeleke, R.A.; et al. Assessing the Mycotoxicological Risk from Consumption of Complementary Foods by Infants and Young Children in Nigeria. *Food Chem. Toxicol.* **2018**, *121*, 37–50,

doi:10.1016/j.fct.2018.08.025.

71. Stanciu, O.; Juan, C.; Miere, D.; Berrada, H.; Loghin, F.; Mañes, J. First Study on Trichothecene and Zearalenone Exposure of the Romanian Population through Wheat-Based Products Consumption. *Food Chem. Toxicol.* **2018**, *121*, 336–342, doi:10.1016/j.fct.2018.09.014.

72. Ciasca, B.; Pascale, M.; Altieri, V.G.; Longobardi, F.; Suman, M.; Catellani, D.; Lattanzio, V.M.T. In-House Validation and Small-Scale Collaborative Study to Evaluate Analytical Performances of Multimycotoxin Screening Methods Based on Liquid Chromatography–High-Resolution Mass Spectrometry: Case Study on Fusarium Toxins in Wheat. *J. Mass Spectrom.* **2018**, *53*, 743–752, doi:10.1002/jms.4089.

73. Puntscher, H.; Kütt, M.L.; Skrinjar, P.; Mikula, H.; Podlech, J.; Fröhlich, J.; Marko, D.; Warth, B. Tracking Emerging Mycotoxins in Food: Development of an LC-MS/MS Method for Free and Modified Alternaria Toxins. *Anal. Bioanal. Chem.* **2018**, *410*, 4481–4494, doi:10.1007/s00216-018-1105-8.

74. Carballo, D.; Font, G.; Ferrer, E.; Berrada, H. Evaluation of Mycotoxin Residues on Ready-to-Eat Food by Chromatographic Methods Coupled to Mass Spectrometry in Tandem. *Toxins (Basel).* **2018**, *10*, doi:10.3390/toxins10060243.

75. Zhao, Z.; Yang, X.; Zhao, X.; Chen, L.; Bai, B.; Zhou, C.; Wang, J. Method Development and Validation for the Analysis of Emerging and Traditional Fusarium Mycotoxins in Pepper, Potato, Tomato, and Cucumber by UPLC-MS/MS. *Food Anal. Methods* **2018**, *11*, 1780–1788, doi:10.1007/s12161-018-1180-7.

76. Huang, X.; Wang, S.; Mao, D.; Miao, S.; Hu, Q.; Ji, S. Optimized QuEChERS Method Combined with UHPLC-MS/MS for the Simultaneous Determination of 15 Mycotoxins in Liquorice. *J. AOAC Int.* **2018**, *101*, 633–642, doi:10.5740/jaoacint.17-0365.

77. Adekoya, I.; Obadina, A.; Adaku, C.C.; De Boevre, M.; Okoth, S.; De Saeger, S.; Njobeh, P. Mycobiota and Co-Occurrence of Mycotoxins in South African Maize-Based Opaque Beer. *Int. J. Food Microbiol.* **2018**, *270*, 22–30, doi:10.1016/j.ijfoodmicro.2018.02.001.

78. Majeed, S.; De Boevre, M.; De Saeger, S.; Rauf, W.; Tawab, A.; Fazal-e-Habib; Rahman, M.; Iqbal, M. Multiple Mycotoxins in Rice: Occurrence and Health Risk Assessment in Children and Adults of Punjab, Pakistan. *Toxins (Basel).* **2018**, *10*, 1–30, doi:10.3390/toxins10020077.

79. Rosa Seus Arraché, E.; Fontes, M.R.V.; Garda Buffon, J.; Badiale-Furlong, E. Trichothecenes in Wheat: Methodology, Occurrence and Human Exposure Risk.

J. Cereal Sci. **2018**, *82*, 129–137, doi:10.1016/j.jcs.2018.05.015.

80. Ingenbleek, L.; Sulyok, M.; Adegboye, A.; Hossou, S.E.; Koné, A.Z.; Oyedele, A.D.; Kisito, C.S.K.J.; Dembélé, Y.K.; Eyangoh, S.; Verger, P.; et al. Regional Sub-Saharan Africa Total Diet Study in Benin, Cameroon, Mali and Nigeria Reveals the Presence of 164 Mycotoxins and Other Secondary Metabolites in Foods. *Toxins (Basel).* **2019**, *11*, 1–24, doi:10.3390/toxins11010054.
81. Ayeni, K.I.; Sulyok, M.; Krska, R.; Warth, B.; Ezekiel, C.N. Mycotoxins in Complementary Foods Consumed by Infants and Young Children within the First 18 Months of Life. *Food Control* **2023**, *144*, 109328, doi:10.1016/j.foodcont.2022.109328.
82. Ezekiel, C.N.; Abia, W.A.; Braun, D.; Šarkanj, B.; Ayeni, K.I.; Oyedele, O.A.; Michael-Chikezie, E.C.; Ezekiel, V.C.; Mark, B.N.; Ahuchaogu, C.P.; et al. Mycotoxin Exposure Biomonitoring in Breastfed and Non-Exclusively Breastfed Nigerian Children. *Environ. Int.* **2022**, *158*, doi:10.1016/j.envint.2021.106996.
83. Delgado-Povedano, M. del M.; Lara, F.J.; Gámiz-Gracia, L.; García-Campaña, A.M. Non-Aqueous Capillary Electrophoresis–Time of Flight Mass Spectrometry Method to Determine Emerging Mycotoxins. *Talanta* **2023**, *253*, doi:10.1016/j.talanta.2022.123946.
84. Zhou, H.; Xu, A.; Liu, M.; Yan, Z.; Qin, L.; Liu, H.; Wu, A.; Liu, N. Mycotoxins in Wheat Flours Marketed in Shanghai, China: Occurrence and Dietary Risk Assessment. *Toxins (Basel).* **2022**, *14*, 1–14, doi:10.3390/toxins14110748.
85. Luo, D.; Guan, J.; Dong, H.; Chen, J.; Liang, M.; Zhou, C.; Xian, Y.; Xu, X. Simultaneous Determination of Twelve Mycotoxins in Edible Oil, Soy Sauce and Bean Sauce by PRiME HLB Solid Phase Extraction Combined with HPLC-Orbitrap HRMS. *Front. Nutr.* **2022**, *9*, doi:10.3389/fnut.2022.1001671.
86. Junsai, T.; Poapolathep, S.; Sutjarit, S.; Giorgi, M.; Zhang, Z.; Logrieco, A.F.; Li, P.; Poapolathep, A. Determination of Multiple Mycotoxins and Their Natural Occurrence in Edible Vegetable Oils Using Liquid Chromatography–Tandem Mass Spectrometry. *Foods* **2021**, *10*, doi:10.3390/foods10112795.
87. De Berardis, S.; De Paola, E.L.; Montevecchi, G.; Garbini, D.; Masino, F.; Antonelli, A.; Melucci, D. Determination of Four Alternaria Alternata Mycotoxins by QuEChERS Approach Coupled with Liquid Chromatography-Tandem Mass Spectrometry in Tomato-Based and Fruit-Based Products. *Food Res. Int.* **2018**, *106*, 677–685, doi:10.1016/j.foodres.2018.01.032.

88. González-Jartín, J.M.; Rodríguez-Cañás, I.; Alfonso, A.; Sainz, M.J.; Vieytes, M.R.; Gomes, A.; Ramos, I.; Botana, L.M. Multianalyte Method for the Determination of Regulated, Emerging and Modified Mycotoxins in Milk: QuEChERS Extraction Followed by UHPLC–MS/MS Analysis. *Food Chem.* **2021**, *356*, doi:10.1016/j.foodchem.2021.129647.

89. Islam, M.N.; Tabassum, M.; Banik, M.; Daayf, F.; Fernando, W.G.D.; Harris, L.J.; Sura, S.; Wang, X. Grains from Manitoba , Canada. **2021**, 1–14.

90. Bessaire, T.; Ernest, M.; Christinat, N.; Carrères, B.; Panchaud, A.; Badoud, F. High Resolution Mass Spectrometry Workflow for the Analysis of Food Contaminants: Application to Plant Toxins, Mycotoxins and Phytoestrogens in Plant-Based Ingredients. *Food Addit. Contam. - Part A Chem. Anal. Control. Expo. Risk Assess.* **2021**, *38*, 978–996, doi:10.1080/19440049.2021.1902575.

91. Polišenská, I.; Jirsa, O.; Vaculová, K.; Pospíchalová, M.; Wawroszova, S.; Frydrych, J. Fusarium Mycotoxins in Two Hulless Oat and Barley Cultivars Used for Food Purposes. *Foods* **2020**, *9*, 1–13, doi:10.3390/foods9081037.

92. Castañares, E.; Pavicich, M.A.; Dinolfo, M.I.; Moreyra, F.; Stenglein, S.A.; Patriarca, A. Natural Occurrence of Alternaria Mycotoxins in Malting Barley Grains in the Main Producing Region of Argentina. *J. Sci. Food Agric.* **2020**, *100*, 1004–1011, doi:10.1002/jsfa.10101.

93. Orlando, B.; Grignon, G.; Vitry, C.; Kashefifard, K.; Valade, R. Fusarium Species and Enniatin Mycotoxins in Wheat, Durum Wheat, Triticale and Barley Harvested in France. *Mycotoxin Res.* **2019**, *35*, 369–380, doi:10.1007/s12550-019-00363-x.

94. Jajić, I.; Dudaš, T.; Krstović, S.; Krska, R.; Sulyok, M.; Bagi, F.; Savić, Z.; Guljaš, D.; Stankov, A. Emerging Fusarium Mycotoxins Fusaproliferin, Beauvericin, Enniatins, and Moniliformin in Serbian Maize. *Toxins (Basel).* **2019**, *11*, 1–14, doi:10.3390/toxins11060357.

95. Ji, X.; Deng, T.; Xiao, Y.; Jin, C.; Lyu, W.; Wang, W.; Tang, B.; Wu, Z.; Yang, H. Evaluation of Alternaria Toxins in Fruits, Vegetables and Their Derivatives Marketed in China Using a QuEChERS Method Coupled with Ultra-High Performance Liquid Chromatography-Tandem Mass Spectrometry: Analytical Methods and Occurrence. *Food Control* **2023**, *147*, 109563, doi:10.1016/j.foodcont.2022.109563.

96. Mohammed, A.; Bekeko, Z.; Yusufe, M.; Sulyok, M.; Krska, R. Fungal Species and Multi-Mycotoxin Associated with Post-Harvest Sorghum (Sorghum Bicolor

(L.) Moench) Grain in Eastern Ethiopia. *Toxins (Basel).* **2022**, *14*, 1–18, doi:10.3390/toxins14070473.

97. Rodríguez-Cañás, I.; González-Jartín, J.M.; Alvariño, R.; Alfonso, A.; Vieytes, M.R.; Botana, L.M. Detection of Mycotoxins in Cheese Using an Optimized Analytical Method Based on a QuEChERS Extraction and UHPLC-MS/MS Quantification. *Food Chem.* **2023**, *408*, doi:10.1016/j.foodchem.2022.135182.

98. El Jai, A.; Zinedine, A.; Juan-García, A.; Mañes, J.; Etahiri, S.; Juan, C. Occurrence of Free and Conjugated Mycotoxins in Aromatic and Medicinal Plants and Dietary Exposure Assessment in the Moroccan Population. *Toxins (Basel).* **2021**, *13*, doi:10.3390/TOXINS13020125.

99. Braun, D.; Ezekiel, C.N.; Marko, D.; Warth, B. Exposure to Mycotoxin-Mixtures via Breast Milk: An Ultra-Sensitive LC-MS/MS Biomonitoring Approach. *Front. Chem.* **2020**, *8*, doi:10.3389/fchem.2020.00423.

100. Hanvi, D.M.; Lawson-Evi, P.; De Boevre, M.; Goto, C.E.; De Saeger, S.; Eklu-Gadegbeku, K. Natural Occurrence of Mycotoxins in Maize and Sorghum in Togo. *Mycotoxin Res.* **2019**, *35*, 321–327, doi:10.1007/s12550-019-00351-1.

101. Kosicki, R.; Twaruzek, M.; Dopierała, P.; Rudzki, B.; Grajewski, J. Occurrence of Mycotoxins in Winter Rye Varieties Cultivated in Poland (2017 2019). *Toxins (Basel).* **2020**, *12*, doi:10.3390/toxins12060423.

102. Zhao, Y.; Zeng, R.; Wang, Q.; Chen, P.; Liu, X.; Wang, X. Aflatoxin B1 and sterigmatocystin: Method development and occurrence in tea. *Food Additives & Contaminants: Part B* **2021**, *15*, 31–37.

103. Yoshinari, T.; Takeuchi, H.; Kosugi, M.; Taniguchi, M.; Waki, M.; Hashiguchi, S.; Fujiyoshi, T.; Shichinohe, Y.; Nakajima, M.; Ohnishi, T.; et al. Determination of Sterigmatocystin in Foods in Japan: Method Validation and Occurrence Data. *Food Addit. Contam. - Part A Chem. Anal. Control. Expo. Risk Assess.* **2019**, *36*, 1404–1410, doi:10.1080/19440049.2019.1628359.

104. Zhao, X.; Liu, D.; Yang, X.; Zhang, L.; Yang, M. Detection of Seven Alternaria Toxins in Edible and Medicinal Herbs Using Ultra-High Performance Liquid Chromatography-Tandem Mass Spectrometry. *Food Chem. X* **2022**, *13*, 100186, doi:10.1016/j.fochx.2021.100186.

105. Knutsen, H.K.; Alexander, J.; Barregård, L.; Bignami, M.; Brüschweiler, B.; Ceccatelli, S.; Cottrill, B.; Dinovi, M.; Grasl-Kraupp, B.; Hogstrand, C.; et al. Risks to

Human and Animal Health Related to the Presence of Moniliformin in Food and Feed. *EFSA J.* **2018**, *16*, doi:10.2903/j.efsa.2018.5082

106. RASFF (Rapid Alert System for Food and Feed). Alternariol and tenuazonic acid in tomato preparation from Turkey via Germany. Reference: 2022.0846.

107. RASFF (Rapid Alert System for Food and Feed). Alternariol and tenuazonic acid in strained tomatoes from Italy. Reference: 2021.6352
